# Mechanism of action deconvolution of the small-molecule pathological tau aggregation inhibitor Anle138b

**DOI:** 10.1186/s13195-023-01182-0

**Published:** 2023-03-14

**Authors:** Layla Hosseini-Gerami, Elena Ficulle, Neil Humphryes-Kirilov, David C. Airey, James Scherschel, Sarubini Kananathan, Brian J. Eastwood, Suchira Bose, David A. Collier, Emma Laing, David Evans, Howard Broughton, Andreas Bender

**Affiliations:** 1 Centre for Molecular Informatics, Yusuf Hamied Department of Chemistry, University of Cambridge, Cambridge, UK; 2 AbsoluteAi Ltd, London, UK; 3 Eli Lilly and Company, Windlesham, UK; 4 Zifo RnD Solutions, London, UK; 5 C4X Discovery, Manchester, UK; 6 Eli Lilly and Company, Corporate Centre, Indianapolis, IN USA; 7 Eli Lilly and Company, Bracknell, UK; 8 Eli Lilly and Company (Retired), Bracknell, UK; 9 Social, Genetic and Developmental Psychiatry Centre, IoPPN, Kings’s College London and Genetic and Genomic Consulting Ltd, Farnham, UK; 10 GSK, Stevenage, UK; 11 DeepMind, London, UK; 12 Eli Lilly and Company, Centro de Inovación, Alcobendas, Spain

**Keywords:** Transcriptomics, Bioinformatics, Machine learning, Network biology, Tau

## Abstract

**Background:**

A key histopathological hallmark of Alzheimer’s disease (AD) is the presence of neurofibrillary tangles of aggregated microtubule-associated protein tau in neurons. Anle138b is a small molecule which has previously shown efficacy in mice in reducing tau aggregates and rescuing AD disease phenotypes.

**Methods:**

In this work, we employed bioinformatics analysis—including pathway enrichment and causal reasoning—of an in vitro tauopathy model. The model consisted of cultured rat cortical neurons either unseeded or seeded with tau aggregates derived from human AD patients, both of which were treated with Anle138b to generate hypotheses for its mode of action. In parallel, we used a collection of human target prediction models to predict direct targets of Anle138b based on its chemical structure.

**Results:**

Combining the different approaches, we found evidence supporting the hypothesis that the action of Anle138b involves several processes which are key to AD progression, including cholesterol homeostasis and neuroinflammation. On the pathway level, we found significantly enriched pathways related to these two processes including those entitled “Superpathway of cholesterol biosynthesis” and “Granulocyte adhesion and diapedesis”. With causal reasoning, we inferred differential activity of SREBF1/2 (involved in cholesterol regulation) and mediators of the inflammatory response such as NFKB1 and RELA. Notably, our findings were also observed in Anle138b-treated unseeded neurons, meaning that the inferred processes are independent of tau pathology and thus represent the direct action of the compound in the cellular system. Through structure-based ligand-target prediction, we predicted the intracellular cholesterol carrier NPC1 as well as NF-κB subunits as potential targets of Anle138b, with structurally similar compounds in the model training set known to target the same proteins.

**Conclusions:**

This study has generated feasible hypotheses for the potential mechanism of action of Anle138b, which will enable the development of future molecular interventions aiming to reduce tau pathology in AD patients.

**Supplementary Information:**

The online version contains supplementary material available at 10.1186/s13195-023-01182-0.

## Background

Alzheimer’s disease (AD) is a chronic neurodegenerative disease characterised histopathologically by abnormal aggregates of amyloid-β (forming extracellular plaques) and microtubule-associated protein tau (forming intracellular neurofibrillary tangles) in the brain, affecting memory, motor function and cognition [1]. Recent research has focused on the reduction of tau pathology for AD treatment, owing to an increased understanding of the molecular mechanisms involved in the formation of neurofibrillary tangles (NFT) which block nerve synapses and cause neuronal cell death. The formation of neurofibrillary tangles from tau protein is caused by its aberrant hyperphosphorylation, thereby leading to self-aggregation due to alterations in phosphorylation levels due to the action of a number of protein kinases, such as GSK3β, CDK5 and JNK [2]. Other hypotheses for the progression of AD disease pathology include sustained neuroinflammation [3] and dysfunction in cholesterol metabolism [4].

**Fig. 1:**
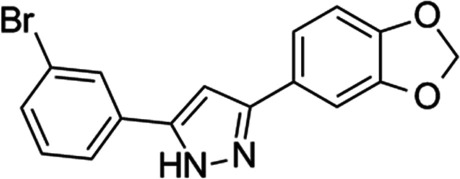
Chemical structure of Anle138b

There are currently no approved drugs which are able to stop or reverse disease progression, with current treatments (cholinesterase inhibitors) focusing on symptomatic relief rather than the underlying disease mechanism of AD [5]. Research in this field has led to the discovery of small molecules which are being investigated due to their ability to inhibit tau aggregation. One such experimental drug is Anle138b (Fig. 1), which effects tau aggregation both in vitro and in vivo [6]. Elucidation of the molecular mechanisms by which Anle138b reduces pathological tau aggregates therefore has the potential to inform new treatments for AD.

The efficacy of Anle138b in the context of AD has been measured in several studies, both in vitro and in vivo, as summarised in Table 1. The seminal paper by Wagner et al. [6] describes its administration in a tau mouse model (PS19) from weaning to terminal disease. They found that Anle138b treatment delayed disease progression, increased survival time and improved cognition and also decreased neuroinflammation and synapse loss. Another study by Brendel et al. investigated the efficacy of Anle138b in a mouse model (involving transgenic mice expressing all 6 hTau isoforms), initiating treatment at a late disease stage (14.5 months of age) rather than from weaning [7]. Metabolic decline was significantly reduced compared to vehicle, and end point tau load, as measured by immunohistochemistry, was correlated with levels of glucose metabolism, indicating that tau pathology is causative of abnormalities in metabolic processes. Martinez Hernandez et al. [8] investigated the effect of Anle138b on amyloid-β channels for rescuing AD disease phenotypes in an amyloid mouse model, showing that it blocks these conducting channels in neuronal membranes, which could be linked to restoration of synaptic plasticity and memory function in vivo. Dominguez-Meijide et al. used a HEK293 cell BiFC (bimolecular fluorescence complementation) model treated with Anle138b to examine its effects on tau aggregation, finding that the compound was not only able to inhibit aggregation but also caused established tau aggregates to disaggregate [9]. The findings from the four studies illustrate the efficacy of Anle138b against Alzheimer’s disease in mice and in vitro, not only by reducing tau aggregation and affecting amyloid-β but also by rescuing disease phenotypes associated with the disease.

**Table 1:** Previous studies of Anle138b efficacy and mechanism of action (MoA) in Alzheimer’s disease

**Study**	**Experimental design**	**Phenotypic observations**	**MoA observations**
Wagner et al. [6]	In vivo*:* Transgenic PS19 mice (frontotemporal dementia model, tau mutation), administration from weaning In vitro*:* (a) Isolated and purified hTau46 cell-free tau aggregation assay (b) Treated primary neurons	Decreased tau aggregation Increased survival time Improved cognition Decreased synapse and neuron loss Reduced hippocampal gliosis and neuroinflammation Decreased tau aggregation None	No change in activity of autophagy markers Reduction in amount of phosphorylated tau Change in fluorescent activity of Anle138b in presence of pre-aggregated tau Slight but not significant increased phosphorylation of GSK3, no change in expression level or activity of PP2A No effect on tau ubiquitination levels
Brendel et al. [7]	Transgenic mice expressing all 6 hTau isoforms, administration at late disease stage	Metabolic decline significantly reduced	Correlation of glucose metabolism with end-point tau load
Martinez Hernandez et al. [8]	*Amyloid*: APPPS1∆9 mice (mouse model for amyloid deposition), treatment both before onset of pathology “pre-plaque”, and after onset of deposition and memory disturbances “post-plaque” *Tau*: Model as in Wagner et al	Rescue of amyloid-induced deficits in synaptic plasticity, long-term potentiation and memory formation None	Binding to and reduction in conductivity of membrane-associated amyloid beta pores No reduction in inflammatory gene expression disease signatures in post-plaque group Amelioration of disease-induced inflammatory gene expression signatures
Dominguez-Meijide et al. [9]	HEK293 venus BiFC (bimolecular fluorescence complementation) cell model treated with Anle138b	Inhibition of the aggregation process and disaggregation of tau aggregates	None

The aforementioned studies into Anle138b efficacy also extended to understanding the mechanism of action through which Anle138b is exerting the observed phenotypic and disease-modifying changes in several instances (right hand column of Table 1). Wagner et al. used an in vitro cell-free tau aggregation assay to identify that Anle138b binds directly to pathological tau aggregates. Moreover, in Anle138b treated murine primary cortical neurons, a small, statistically insignificant, increase in phosphorylated GSK3β, and no change in PP2A (tau phosphatase) protein expression or activity, compared to untreated, was observed. These observations lead to the conclusion that Anle138b does not reduce tau aggregation by modifying these two proteins known to be upstream of tau hyperphosphorylation. However, following their in vivo assays, they extracted brain samples from treated and untreated mice and found a reduction in phosphorylated tau in the treated group. Furthermore, the authors observed unchanged activity of autophagy markers (PS19 mouse brain samples) or ubiquitinated tau (primary neurons). Through transcriptional profiling of pre- and post-plaque mice (amyloid model), Martinez Hernandez et al. found that aberrant gene expression signatures seen in the hippocampus, corresponding to neuroinflammation pathways induced by tau pathology, were significantly ameliorated when comparing Anle138b-treated to vehicle-treated mice. No further analysis into the mechanism of action of Anle138b when administered after the onset of pathology was undertaken by either Wagner et al. or Martinez Hernandez et al., since both studies treated mice with Anle138b from weaning. Hence, although current reports indicate that treatment with Anle138b reduces tau hyperphosphorylation, and ameliorates gene expression signatures associated with neuroinflammation, the mechanisms through which this would occur after disease has been established are unclear.

In this work, we therefore aim to further understand the mechanism of action of Anle138b efficacy on pathological tau aggregates at the protein target, pathway and gene level in an integrated manner, involving both predicted ligand–protein interactions and gene expression changes, providing a more comprehensive view of compound action (for a recent review on ways of analysing and understanding the mode of action of a compound see [10], and for prior integrated studies see [11–13]. Understanding the molecular mechanisms behind the observed reduction in tau phosphorylation and amelioration of neuroinflammation gene signatures observed previously is still of significant interest due to the following: the Wagner in vitro study used a cell-free tau assay to conclude that Anle138b binds directly to tau aggregates, but the lack of cellular context means that they could have missed other protein targets of Anle138b that may be relevant to tau clearance—in fact, it has been suggested that drugs which are able to both directly bind to tau aggregates and interact with its chaperone proteins would be an optimal strategy for Alzheimer’s treatment [14].

Some of the authors of this work recently developed a microfluidic cell model of tau aggregation and propagation (using hAD derived tau seeds to induce a time-dependent increase in endogenous tau inclusions in rat cortical neurons (RCN)) [15] and demonstrated, via multi-omic analysis, the utility of this model to recapitulate relevant AD pathways/processes at the molecular level [16]. Microfluidic cell models have been established in recent years as suitable in vitro models for AD to study neuronal connectivity and the spread of tau pathology [17–23], and they can be used to test intervention (by, e.g. small molecules) against neuronal cell death (characterised by loss of synapses and neuronal network in the cell model), which is one of the main pathophysiological characteristics of the disease [15]. This neuronal model may be a suitable platform for high-throughput screens for target or hit compound identification and validation. When Anle138b was applied to this model, they found via high content imaging that the compound inhibited aggregation (by ~ 50% compared to DMSO control) and propagation (by ~ 40% compared to control) of tau in RCNs seeded with hAD tau [15]. With transcriptomics of seeded and control cells, we identified 1075 differentially expressed genes (including 26 altered at two time points). These were enriched for lipid/steroid metabolism and neuronal/glial cell development genes. Fifty genes were correlated with tau inclusion formation at both transcriptomic and proteomic levels, including several microtubule and cytoskeleton-related proteins such as Tubb2a, Tubb4a, Nefl and Snca. Several genes (such as Fyn kinase and PTBP1, a tau exon 10 repressor) interact directly with or regulate tau.

Here, we use this model to understand how Anle138b is able to interact with cells in a state representative of AD pathology, i.e. seeded with tau. In addition, we carried out parallel experiments on unseeded rat neurons, also treated with Anle138b, to ensure that any findings were not a consequence of a reduction in tau aggregation caused by the drug. For the analysis of relevance of the DEGs to Alzheimer’s disease, we used a set of genes relevant to AD, selected from the Open Targets platform [15] Alzheimer’s Disease and Tauopathies association lists, comprising 5730 and 3622 genes respectively, forming a list of 5991 unique genes included in the analysis. By performing protein–protein interaction causal reasoning and pathway enrichment with the transcriptional data derived from Anle138b-treated RCNs, and target prediction using its chemical structure, we were able to hypothesise several alternative mechanisms of action of Anle138b with evidence across all types of computational analysis.

## Results and discussion

### Experimental and informatics workflow

**Fig. 2:**
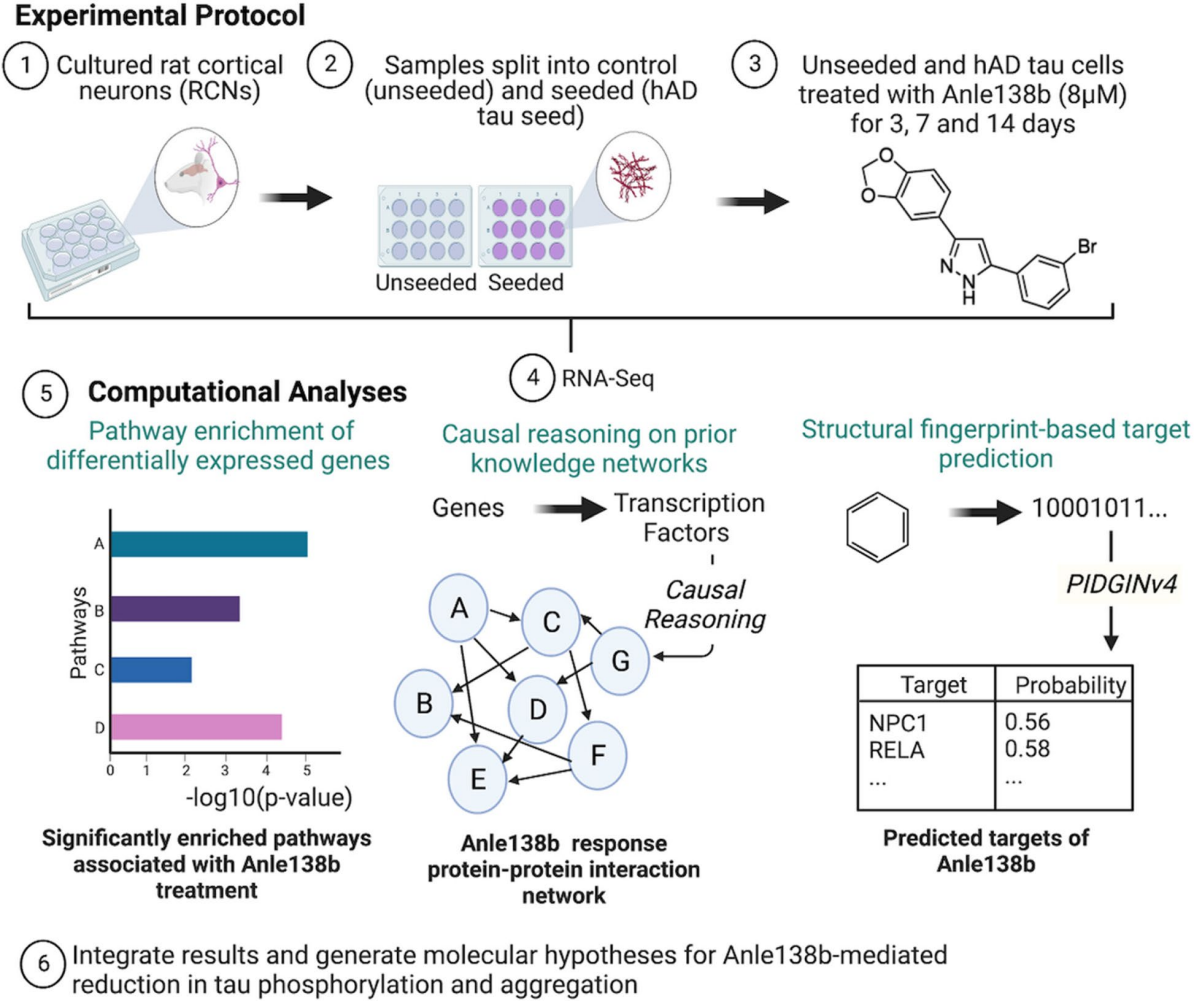
In the current study, (1) rat cortical neurons (RCNs) were used as the cellular basis for investigation and (2) were either seeded with hAD tau or left as unseeded unseeded. Cells were (3) treated with 8 μm Anle138b for three different durations then (4) RNA-Seq experiments were undertaken to measure mRNA abundance. Once data was available, (5) chem- (ligand-target prediction) and bioinformatics analyses (pathway enrichment and causal reasoning) were carried out in parallel, and (6) integrated to generate hypotheses for the mechanism of action of Anle138b. Created with BioRender

The workflow of the current study (including preceding experimental steps) is shown in Fig. 2. Following the culturing of RCNs (step 1) and splitting into two groups (unseeded and hAD tau seeded cells) (step 2), both groups were treated with Anle138b and samples collected after three different treatment durations (step 3) and analysed by RNA-Seq (step 4). Differential expression for unseeded cells treated with Anle138b at each of the three time-points was compared to unseeded cells treated with vehicle, whereas differential expression for seeded cells treated with Anle138b at each of the three time points was compared to seeded cells treated with vehicle. Downstream analysis was performed in parallel using differential pathway analysis, causal reasoning and ligand-target prediction (step 5), and results from the different streams were integrated to generate mode of action hypotheses for Anle138b (step 6).

### Anle138b reverses the expression of genes dysregulated by hTau seed

In a previous study by Ficulle et al., we seeded rat primary cortical neurons with tau to induce a time-dependent increase in endogenous tau inclusions and performed transcriptomic and proteomic analysis between seeded and unseeded cells to identify differentially expressed genes [16]. These differentially expressed genes were enriched for lipid/steroid metabolism and neuronal/glial cell developmental processes. Fifty genes were correlated with tau inclusion formation at both transcriptomic and proteomic levels, including several microtubule and cytoskeleton-related proteins such as Tubb2a, Tubb42a, Nefl (microtubule cytoskeleton organisation) and Snca (actin binding). Using this cellular model, Anle138b was found to inhibit tau aggregation and propagation by 50% and 40%, compared to DMSO control [15]. We compared the differentially expressed transcripts in Ficulle et al. (2022) with the Anle138b-treated vs vehicle control hAD tau RCN differentially expressed transcripts in the present study (Supplementary File 1) at each timepoint (3, 7 and 14 days) to understand if Anle138b is able to “reverse” the expression of genes known to be differentially expressed as a consequence of seed, in terms of their log2FC (Figure S1-S3). We found genes at each time-point that are upregulated as a consequence of seed and downregulated by Anle138b (including Lpar1, Cntnap4, Plp1) and that are downregulated as a consequence of seed and up-regulated by Anle138b (including Ccdc103, Trpc7, Znrf4). From these findings, we concluded that tau amelioration by Anle138b is reflected in its transcriptomic signatures, and hence, the transcriptomic data in this study can be analysed to generate hypotheses for the mechanism of action of Anle138b-induced tau aggregation inhibition. We note that out of all differentially expressed genes in seeded RCNs, only a small number of genes were found to be reversed by Anle138b. However, changes to gene expression are only one layer of biology and do not fully represent the mechanism of action of Anle138b. This is why in the current study we integrated other sources of information, namely pathway annotations, protein–protein interaction and chemical structure, to investigate the MoA of Anle138b on a deeper level.

### Anle138b induces changes in the transcription of known AD genes

**Fig. 3:**
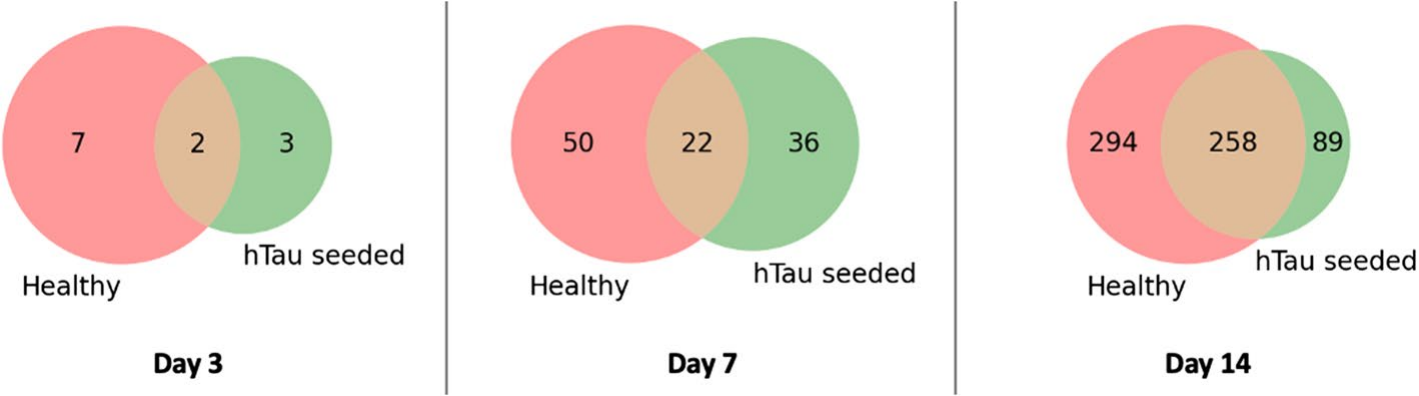
Venn diagrams showing number of DEGs and their overlap between cell conditions (unseeded or hTau seeded) at each time point (3, 7 and 14 days)

In unseeded Anle138b-treated RCNs, there were 9, 72 and 552 differentially expressed genes (DEGs) at days 3,7 and 14, respectively, and in hAD tau seeded Anle138b-treated RCNs, there were 5, 58 and 347 DEGs at the same time points (Fig. 3).

We analysed the DEGs induced by Anle138b treatment in comparison to vehicle control unseeded or hAD tau seeded RCNs at each time point to investigate if the genes modulated by Anle138b treatment were known to be associated with AD in either cellular state. To facilitate this, we identified a set of human genes a priori associated with AD utilising the Open Targets platform [24] Alzheimer’s Disease and Tauopathies association lists, comprising 5730 and 3622 genes respectively, and forming a list of 5991 unique genes.

**Fig. 4:**
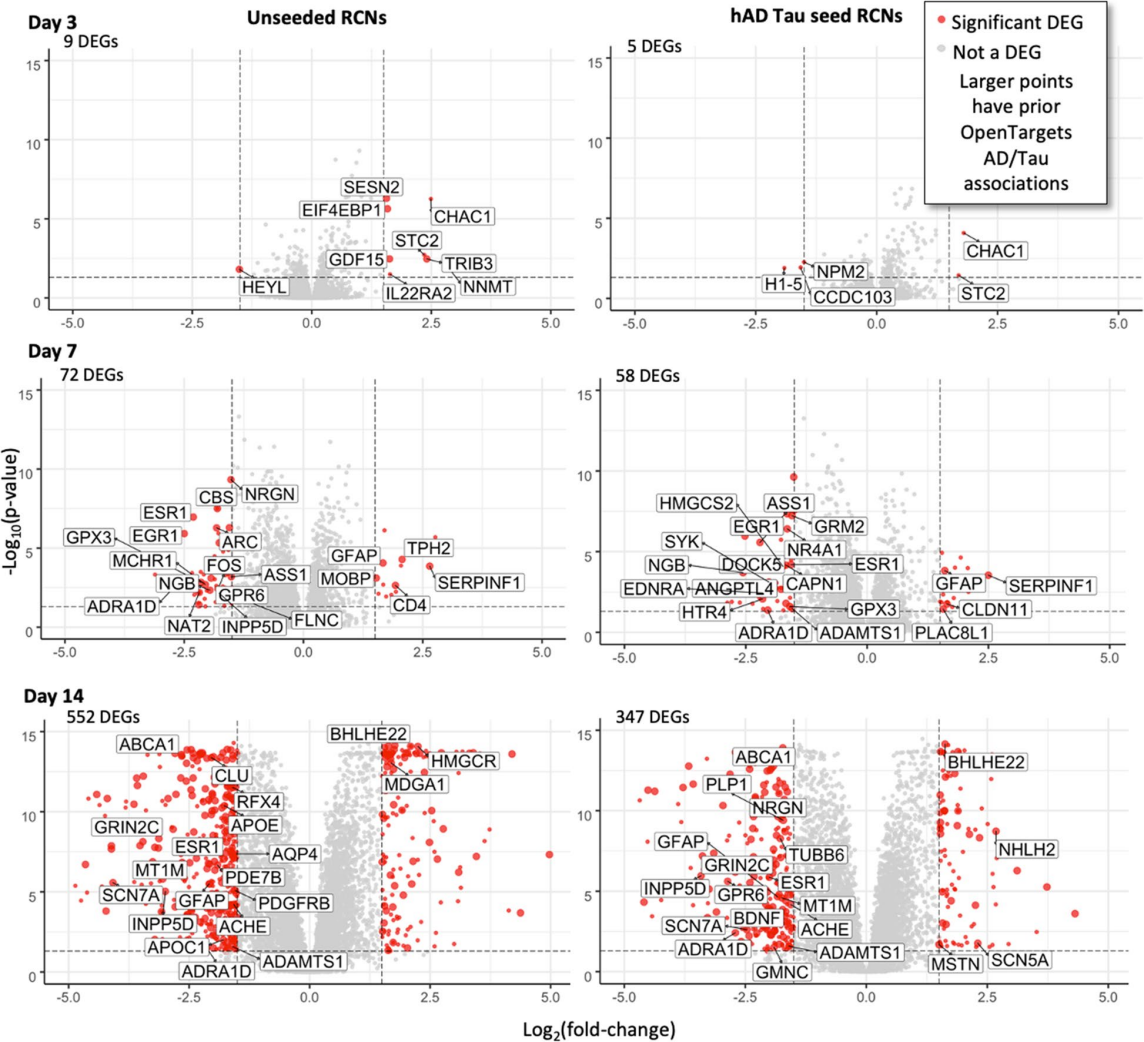
Differentially expressed genes after 3, 7 and 14 days of treatment (in columns) and for unseeded cells (left) and seeded cells (right). Points are coloured by whether they satisfy the threshold of p ≤ 0.05 and |Log2FC |≥ 1.5 (indicated by dashed lines on each plot). DEGs which appear in the Open Targets Alzheimer Disease or Tauopathies association lists are represented by enlarged points (see Supplementary File 2 for full association score details). Genes of interest are labelled if they are either part of the most differentially expressed genes or part of the Open Targets association lists or both

Genes modulated by Anle138b possess a prior known association with AD, according to the data used (Fig. 4, and Supplementary File 2 outlays the full details of association scores of each DEG in each experiment). The overlap between DEGs and AD genes is significant with respect to all measured genes only at day 14, in both unseeded (OR = 1.44, *p* = 0.00017) and hAD tau seeded (OR = 1.28, *p* = 0.022) Anle-treated RCNs according to Fisher’s exact test (Table S1 contains full results). All experiments other than day 3 hAD tau seeded (top right of Fig. 4) identified DEGs associated with AD, with their number increasing with treatment time (see Fig. 4, and Table S1 for details). The protein products of these genes take part in a wide variety of functions such as sodium transport (*SCN5A and SCN7A*, both association scores = 1), lipid regulation (*APOC1*,* APOE*, association scores = 0.87 and 1 respectively) and calcium signalling (*NGRN*,* STC2*, association scores = 0.21 and 1 respectively, and *CAPN1*), as well as GPCR signalling (*GPR6*, *GRM2*,* MCHR1*, association scores = 0.20, 0.04 and 0.27 respectively). Thus, through RNA-Seq profiling of Anle138b-treated hAD tau seeded and unseeded RCNs, we identified significant differential expression of genes with previous literature associations to AD and tauopathies. Together, these findings led us to hypothesise that, in addition to direct aggregate binding, Anle138b also inhibits tau aggregation via additional molecular mechanisms.

### Anle138b modulates pathways related to cholesterol metabolism, inflammation and extracellular matrix organisation

The first analysis of the Anle138b-induced DEGs in the seeded and unseeded RCNs was pathway enrichment, to contextualise the genes and understand which downstream processes may be affected by Anle138b treatment. For our pathway knowledgebase, we aimed for coverage of diverse biological processes by combining GO, Reactome, IPA and MetaCore databases (see *Materials and Methods* for details). To understand the impact of hAD tau seeding as well as duration of Anle138b treatment on differentially expressed genes, we first identified commonly dysregulated pathways in both hAD tau and unseeded Anle138b-treated RCNs at each time point (enrichment indicated by an FDR-adjusted *p*-value ≤ 0.05 in a pair of experiments). We additionally calculated overlap coefficients [25], to understand how the number of overlapping pathways related to the number of significantly enriched pathways obtained overall for each experiment (defined as the size of the intersection divided by the smallest set size), ranging from 0 to 1. These results are summarised with the higher-level processes described by the pathways and their relevance to AD in Table 2.

**Table 2:** Number of overlapping pathways and their overlap coefficient between the different Anle138b experiment pathway enrichment results (FDR-adjusted *p*-value ≤ 0.05), a summary of high-level processes described by the pathways and their relevance to AD

**Experiment 1**	**Experiment 2**	**Number of shared significant pathways (overlap coefficient)**	**Summary of processes**	**Relevance to AD**
hAD Tau seeded RCNs, day 14	Unseeded RCNs, day 14	47 (0.59)	GPCR signalling Extracellular matrix (ECM) organisation Cholesterol homeostasis Inflammation Angiogenesis	Mediates both the inflammatory response in microglia and modulation of calcium signalling [26, 27] Reduction in hippocampal ECM reverses memory deficits associated with AD [28] Regulates both tau and amyloid pathology by independent mechanisms [29] Causative of and aggravated by tau pathology [30] Tau pathology induces blood vessel abnormalities [31]
hAD Tau seeded RCNs, day 7	Unseeded RCNs, day 7	4 (0.29)	Metabolism	Disruptions in metabolic processes linked to AD progression [32–34]
hAD Tau seeded RCNs, day 3	Unseeded RCNs, day 3	0 (0.00)	-	-

This analysis shows a high overlap between dysregulated pathways associated with Anle138b treatment at day 14 between hAD tau seeded RCN and unseeded RCN experiments (of 47 pathways with an overlap coefficient of 0.59, Table 2). All overlapping pathways can be found in Table S2, and all enriched pathways and their associated adjusted *p*-values can be found in Supplementary File 3. The pathways dysregulated after Anle138b treatment both in the absence and presence of tau pathology at day 14 (Table S2) relate to GPCR signalling (e.g. “Phospholipase C-activating G-protein coupled receptor signalling”), ECM matrix organisation (e.g. “Degradation of the extracellular matrix”), cholesterol homeostasis (e.g. “Superpathway of cholesterol biosynthesis”), inflammation (e.g. “Granulocyte adhesion and diapedesis”) and angiogenesis (e.g. “endothelial cell proliferation”). The role of GPCRs in AD have been well characterised, mediating both the inflammatory response in microglia and modulation of calcium signalling [26, 27]. Mouse models have shown that pharmacological reduction in hippocampal extracellular matrix has reversed early memory deficits associated with AD, restoring both long-term potentiation and memory performance [28] which were shown to be restored with Anle138b in previous in vivo studies [8]. Previous pathway analyses on differential genes in AD vs control temporal cortex samples by Morabito et al. revealed, in agreement with our results, an upregulation of extracellular matrix organisation genes [35], and ECM pathways enriched in a module identified in genetic networks created from AD genes differentially expressed in the blood [36]. Van der Kant et al. identified that cholesterol metabolism is a ‘druggable axis’ which regulates both tau and amyloid pathology by independent mechanisms [29]. Neuroinflammation gene signatures have been found to be ameliorated by Anle138b before [8], and neuroinflammation in general is known to be both causative of and aggravated by tau pathology in AD [30]. In the context of previous studies, “Granulocyte adhesion and diapedesis” (a process in which white blood cells move to a site of inflammation) pathway was previously found in Hu et al.’s network and pathway-based analysis of AD genes [37], whilst “leukocyte migration” was significantly enriched in upregulated genes found in the aforementioned Morabito et al. AD vs control temporal cortex samples [35]. Finally, tau NFT formation induces abnormalities in blood vessels and the expression of angiogenesis-related genes [31]. Thus, pathways enriched at day 14 following Anle138b-treatment in both the presence (providing disease context) and absence (ensuring findings are not solely due to reduction in tau aggregates) of hAD tau seed encompass processes known to be crucial to AD and tau pathology, including processes which have been previously found to be modulated in previous Anle138b studies and identified from prior bioinformatics analyses of AD disease models and genes.

Pathways found at day 7 associated with Anle138b treatment in the presence and absence of hAD tau seed (4 pathways, overlap coefficient = 0.29, Table 2) were metabolic in nature, including “Glutathione redox reactions” (Table S2). Hu et al.’s AD gene analysis also revealed a significant enrichment of the “Glutathione metabolism” pathway [37]. Disruptions in metabolic processes in general, including the other pathways found at this time point which come under the umbrella of the urea cycle, has been linked to AD progression [32–34]. Interestingly, phenylbutyrate—a drug used to treat urea cycle disorders—reverses tau pathology and behavioural deficits in a mouse model of AD [38], much like Anle138b [6]. This indicates that Anle138b could be modulating metabolic processes at day 7 of treatment—previous experiments have shown that the compound restores metabolic decline in mice after late-stage AD treatment [7].

There were no significantly enriched pathways identified at day 3 in both hAD tau seeded and unseeded RCNs. Pathways enriched at day 3 in unseeded RCNs included “IL-22 signalling” (FDR-adjusted *p*-value = 0.018), “Response to endoplasmic reticulum stress” (FDR-adjusted *p*-value = 0.003) and “PI3K/AKT signalling” (FDR-adjusted *p*-value = 0.004) (Figure S3). IL-22 is produced by Th17 cells, which are secreted in the presence of Amyloid-b pathology in microglia [39]. Endoplasmic reticulum (ER) stress has been linked to AD phenotypes due to the accumulation of pathogenic misfolded proteins and perturbations to intracellular calcium signalling [40]. PI3K/AKT signalling, linked to insulin signalling, is altered in the AD brain, and this signalling cascade inhibits the activity of GSK3b—a key kinase in tau hyperphosphorylation [41]. Only two pathways were significantly enriched at day 3 in hAD tau seeded RCNs following Anle138b treatment (Figure S4), “Glutathione metabolism” (FDR-adjusted *p*-value = 0.01) and “HIF-1 targets” (FDR-adjusted *p*-value = 0.02). It has been found that glutathione (an intracellular antioxidant) is reduced in human AD pathology [42] and hypoxia-inducible factor-1 (HIF-1) target genes are involved in angiogenesis, glucose metabolism and cell proliferation. In particular, the downstream gene VEGFR is found to be altered in AD, and experimental evidence has indicated that regulating HIF-1 may alter cellular and tissue damage in neurodegenerative diseases [43]. This shows that processes relevant to AD progression are being modulated at an early stage of Anle138b treatment.

### Anle138b transcriptional response networks inferred from causal reasoning include ubiquitination proteins and mediators of cholesterol homeostasis

**Fig. 5:**
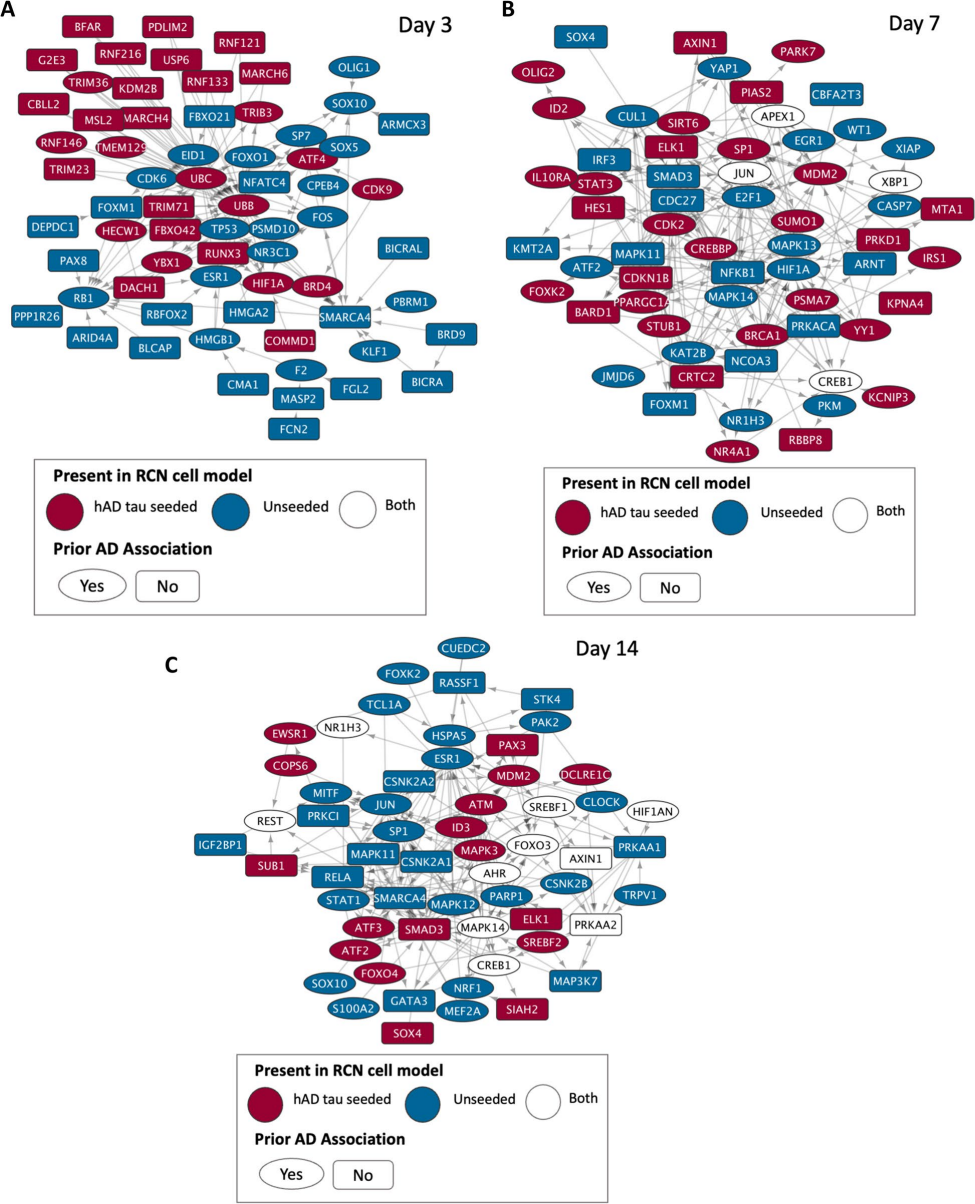
Reconstructed signalling networks of protein–protein interactions from causal reasoning analysis of the transcriptional response of unseeded and hAD tau seeded RCNs treated with Anle138b, at all time points. Nodes (proteins) in the network are connected by edges with arrows representing their known directed protein–protein interaction from the Metabase™ network

The second analysis undertaken with the profiled transcripts was causal reasoning, to identify the upstream protein regulators responsible for the observed transcriptomic response to Anle138b treatment based on prior knowledge of protein–protein interactions (PPI). This analysis led us to reconstruct subnetworks of inferred perturbed proteins for each experiment. To this end, we pooled results from each causal reasoning algorithm used across different prior knowledge networks and reconstructed networks at each time point from the regulators which were recovered most frequently across all analysis setups (illustrated in Fig. 5 and tabulated in Table S3).

The day 3 network included genes involved in protein ubiquitination (PSMD10, UBB, UBC, USP6, MARCHF6, FBXO21, HECW1, RNF216, RNF121, RNF146, TMEM129, TRIM71, COMMD1, RNF133, TRIM36), a process known to be associated with the disease [44] (Fig. 5A). Wagner et al. studied Anle138b changes to markers of proteasomal tau degradation and tau ubiquitination, finding no change in ubiquitinated tau protein levels and no degradation of tau when Anle138b was administered with translation inhibitor cycloheximide [6]. However, they carried out their findings in primary neurons with no AD pathology present, and our protein ubiquitination nodes were mainly seen in the network reconstructed from the transcriptomic data in hAD tau seeded RCNs (red nodes in Fig. 4). Therefore, it would be useful to repeat Wagner et al.’s ubiquitination and proteasomal marker analysis using hAD tau seeded RCNs to investigate Anle138b’s effects on tau ubiquitination and proteasomal degradation in the presence of cellular tau aggregates. ATF4 was recovered across 8 different combinations of prior knowledge networks/algorithms and is involved in the unfolded protein response as well as mediating processes such as autophagy, oxidative stress and inflammation and is thus credibly implicated in neurodegeneration and AD [45].

The network reconstructed at day 7 contains nodes implicated in glucose metabolism (HIF1A, FOXK2, IRS1, PPARGC1A, SIRT6, PKM) (Fig. 5B). Anle138b is already known to have an effect on glucose metabolism, restoring normal metabolism in a mouse model of late-stage AD [7] and also halting AD progression [33]; therefore, our results are consistent with previous knowledge in this regard. Additionally, ELK1 was recovered, and although not annotated in Open Targets as AD- or tau-related, it was found to inhibit the transcription of PS1 which is involved in the proteolytic processing of amyloid precursor protein into amyloid plaques [46], and it hence also represents a plausible contribution to the mode of action of Anle138b.

The day 14 network contained a number of genes related to cholesterol homeostasis (NR1H3, SREBF1 in unseeded and hAD tau seeded RCN, SREBF2 in hAD tau seeded RCN only) (Fig. 5C). Additionally, TRPV1 was found in the unseeded RCN network, which is activated by inflammatory molecules and involved in calcium homeostasis [47] and also mediates synaptic long-term depression which was modulated by Anle138b [8]. ELK1 was again recovered, as well as STAT1, which is a transcription factor with a key role in neuroinflammation, spatial learning and memory formation [48].

Overall, this analysis enabled us to identify which signalling proteins could be modulated by Anle138b treatment over the duration of 3-, 7- and 14-day treatment on the expression level and to relate them to previous phenotypic findings such as glucose metabolism and inflammation. We additionally inferred the differential activity of proteins involved in ubiquitination and cholesterol homeostasis, which are both associated with tau pathology and AD. Though some nodes in the networks do not have prior associations with AD, they could still be relevant to the mechanism of action of Anle138b against tau aggregation, due to the fact they directly interact with known AD proteins.

### Target prediction of Anle138b highlights known AD targets and those functionally related to Anle138b phenotypes

In addition to bioinformatics analyses, we utilised the chemoinformatics target prediction tool PIDGINv4 [49] on the chemical structure of Anle138b to identify potential direct interaction partners of the compound. We report the top 10 predicted targets based on predicted probability of activity at the stated threshold of 1, 10 and 100 μM and with applicability domain (measure of prediction confidence, see [50]) percentile ≥ 50 (Table 3, all predictions with a probability > 0.3 can be found in Table S4; for the full matrix of predictions, see Supplementary File 5).

**Table 3:** Ten predicted protein targets of Anle138 based on chemical structure-based target prediction with highest activity likelihood (given in ‘probability’ column). Each prediction has an associated applicability domain (ad) estimate from 0 to 100 to infer the confidence in the prediction (with 100 being highest confidence). Also given is the “nearest neighbour” (most structurally similar compound to Anle138b in the training set of each model), with the associated Tanimoto similarity based on their ECFP4 fingerprints. Each target is also annotated with its association with the Open Targets Alzheimer’s Disease (AD) or Tauopathies (Tau) lists

**Gene symbol**	**Activity threshold (μ**M**)**	**Probability**	**ad**	**Nearest neighbour to Anle138b**	**Tanimoto similarity**	**AD Gene**	**Tau gene**
**NFKB2**	10 100 1	0.62 0.61 0.51	72 72 75	CHEMBL1567097	0.50	N	N
**ALOX15**	10 100 1	0.60 0.46 0.31	66 72 61	CHEMBL1567097 CHEMBL239677	0.50 0.23	Y	Y
**SENP8**	100 10	0.59 0.40	76 82	CHEMBL1370387	0.45	N	N
**RELA**	1 100 10	0.58 0.52 0.42	71 69 69	CHEMBL1567097	0.50	N	N
**RAB9A**	100	0.58	57	CHEMBL1567097	0.50	Y	N
**NPC1**	100	0.56	58	CHEMBL1567097	0.50	Y	Y
**SENP6**	100	0.56	79	CHEMBL1370387	0.45	N	N
**NFKB1**	1 10 100	0.52 0.52 0.52	73 68 68	CHEMBL1567097	0.50	N	N
**ATAD5**	100	0.52	99	CHEMBL1567097	0.50	N	N
**CLK1**	10 1 100 0.1	0.52 0.51 0.45 0.44	77 91 77 82	CHEMBL2392365	0.41	Y	Y

The highest predicted targets comprise four proteins with prior AD associations, namely ALOX15, RAB9A, NPC1, and CLK1 (Table 3). It has been established before that mice treated with a selective inhibitor of ALOX15 (probability = 0.60 @ 10 μM, ad = 66) showed a significant reduction in amyloid and tau pathology—including tau phosphorylation via CDK5 and SAPK/JNK modulation—as well as restoring memory and synaptic integrity [49], another phenotypic effect of Anle138b [8]. RAB9A (probability = 0.58 @ 100 μM, ad = 57) plays a role in autophagy, a process which degrades misfolded and aggregated proteins such as tau and a-synuclein [51], which was also ameliorated by Anle138b treatment [52]. NPC1 (probability = 0.56 @ 100 μM, ad = 58) mediates intracellular cholesterol trafficking and cytosolic tau entry [53], and disease of this protein (Niemann-Pick disease, type C1) is also associated with intraneuronal tau NFTs [54]. CLK1 (probability = 0.52 @ 10 μM, ad = 77 and 0.51 @ 1 μM, ad = 91) has been suggested as a target for alleviating AD, as it regulates alternative splicing of tau protein [55].

Further to the proteins with prior established AD associations, nuclear factor-kappa B subunits (e.g. NFKB2, probability = 0.62 @ 10 μM, ad = 72 as well as RELA and NFKB1) appeared in the top 10 predictions (Table 3). Though NFKB2 does not appear in the Open Targets association list, NF-kB plays a role in AD progression [56], with modulations in its signalling pathway triggering neuroinflammation, oxidative stress and cell death, whilst on the other hand contributions to the maintenance of synaptic plasticity and memory function have been previously established [57]. Additionally, sentrin-specific proteases (e.g. SENP8, probability = 0.59 @ 100 μM, ad = 76 as well as SENP6) are involved in ubiquitination, a process which is able to ameliorate tau pathology through degradation [44].

We next looked at structurally similar compounds in the set of compounds that these models were trained on, which is useful to rationalise our predictions and to evaluate their plausibility. The nearest neighbours to Anle138b have a Tanimoto similarity of 0.5 and 0.45 and are experimental drugs, represented in the ChEMBL database as CHEMBL1567097 and CHEMBL1370387, respectively.

**Fig. 6:**
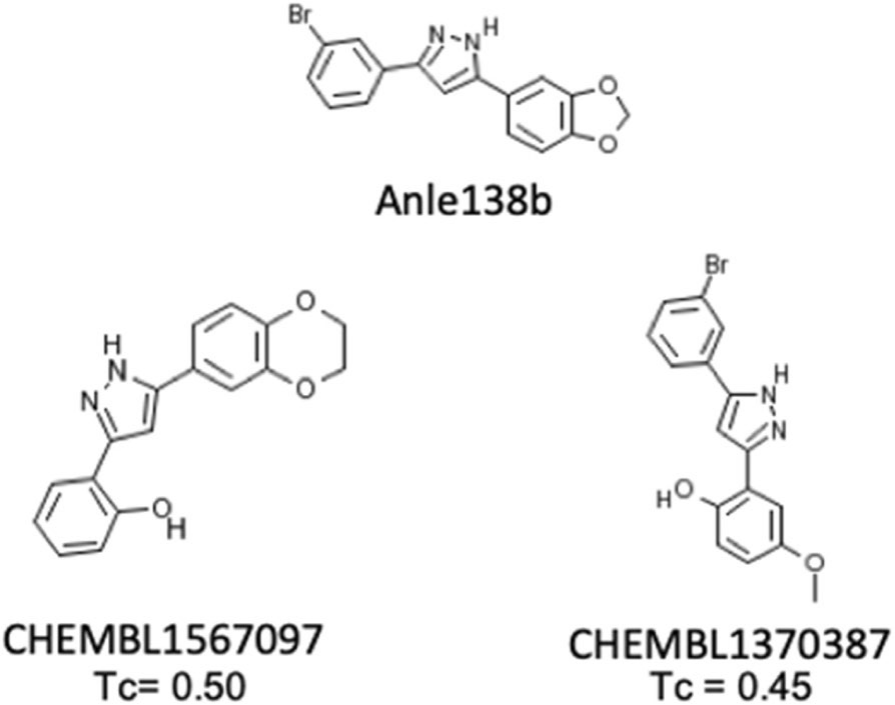
Two nearest neighbours to Anle138b active against predicted targets in the PIDGINv4 training set, based on the Tanimoto similarity (Tc) of ECFP4 fingerprints

CHEMBL1567097 is very chemically similar to Anle138b, with the only difference being a change from the meta-Bromine to an ortho-hydroxyl group on the benzene moiety, and an extension of the 5- to a 6-membered ring (Fig. 6). Therefore, we consulted with the ChEMBL and PubChem databases to understand the mechanism of action of the compound (Table 4). CHEMBL1567097 is active against NPC1, RAB9A (sub-micromolar) and ALOX15 (12.6 μM), as well as showing inhibition of tau fibril formation at 20 μM, whilst being inactive against tau filament binding, and it increases the expression of NF-kB at 0.64 μM [58]. Notably, it has been patented as a treatment for neurodegenerative diseases such as AD [59].

**Table 4:** Known activities of CHEMBL1567079 and CHEMBL1370387, nearest neighbour to Anle138b, data retrieved from ChEMBL and PubChem

**Compound**	**Target/process**	**Activity (μM)**
CHEMBL1567097	NPC1 activation	0.50
	Increased expression of NF-kB in neuronal cells	0.64
	RAB9A activation	0.71
	ALOX15 inhibition	12.6
	Inhibition of tau fibril formation	20.0
	Tau filament binding	Inactive [60]
CHEMBL1370387	RAB9A activation	1.27
	NPC1 activation	5.62
	ALOX15 inhibition	7.00
	Inhibition of tau fibril formation	10.0
	Alpha-synuclein inhibition	14.1
	SENP6 inhibition	26.5
	SENP8 inhibition	29.1
	Tau filament binding	Inactive [60]

CHEMBL1370387 is also very structurally similar to Anle138b, but with an opening of the dioxolane ring, forming ortho-hydroxyl and meta-ether moieties on the benzene ring (Fig. 6). It also shows activity (< 10 μM) towards RAB9A, NPC1 and ALOX15 and inhibits tau fibril formation at 10 μM. It also inhibits alpha-synuclein at 14.1 μM, and Anle138b additionally showed amelioration of alpha-synuclein pathology in in vivo models of Parkinson’s disease [61]. CHEMBL1370387 is also active against predicted targets of Anle138b SENP6 and SENP8 at 26.5 and 29.1 μM, respectively. It is inactive against tau filament binding (Table 4). Due to the high structural similarities with Anle138b, and as these compounds were also found to inhibit tau aggregation, this provides added confidence in the findings of the aforementioned targets.

Hence, any of these protein targets are plausible mode of action hypotheses for Anle138b as their modulation would potentially lead to reduced tau aggregation and additional phenotypic effects that were observed in previous studies. It appears on the other hand likely that not one precise target will be able to explain the mechanism of Anle138b, as is evident from the multitude of changes on the gene expression level (discussed before), as well as the extensive list of direct protein interactors shown here.

### Integration of findings on the target, signalling protein, pathway and gene-level enables the generation of detailed hypotheses for Anle138b’s mechanism of action

**Fig. 7:**
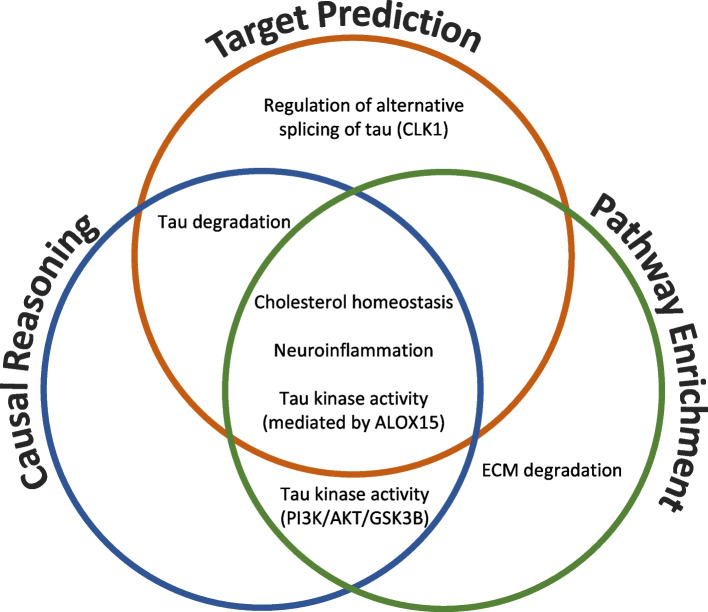
Biological processes predicted to be modulated by Anle138b based on the results from gene expression data analysis using causal reasoning and pathway enrichment and ligand-target prediction. This analysis hence provides an integrated view on the putative mode of action of this compound, from different angles

Given that direct protein interactors of a compound, as well as downstream gene expression changes (and the different enrichment and causal reasoning methods applied) provide a different angle to the mode of action of Anle138b, we next integrated the results from each method to provide an integrated view. In this case, we chose to focus on the *overlap* between methods, since here increased confidence (consistent signal) is obtained on different levels, and since practically the union of all results would be rather comprehensive and much more difficult to interpret in detail. The main mechanistic hypotheses generated from the analyses were based on the following criteria: (a) evidence from at least one of the three methods, (b) would feasibly lead to amelioration of tau pathology based on prior literature and (c) would correspond with prior Anle138b phenotypic observations (Fig. 7).

It can be seen that cholesterol homeostasis, neuroinflammation and tau kinase activity (mediated by ALOX15) were processes which were appearing in all three analyses (target prediction, causal reasoning and pathway enrichment). Evidence suggesting tau degradation by ubiquitin/proteasomal system or autophagy were found from target prediction and causal reasoning and tau kinase activity (via the PI3K/AKT/GSK3b signalling cascade) was identified from causal reasoning and pathway enrichment. These results show the utility of computational analysis of biological and chemical data for generating testable hypotheses for compound mechanism of action, particularly in combination with prior phenotypic knowledge. We will now expand on the targets and processes included in Fig. 7 in more detail.

*Mediation of cholesterol homeostasis* was observed in all three analyses. Predicted target NPC1 (Table 3, probability = 0.56) is involved in intracellular cholesterol trafficking. NPC1 when overexpressed has been found to significantly reduce seeded aggregation by reducing cytosolic tau assembly entry via an increase in membrane cholesterol levels (the reverse was seen following NPC1 knockdown) [53]; hence, pharmacological activation of NPC1 may lead to similar findings. Although the chemical-structure based target prediction performed in this study does not infer directionality (i.e. activation or inhibition of the target), NPC1 is known to be activated by structurally similar compounds CHEMBL1567097 and CHEMBL1370387 at 0.5 and 5.62 μM, respectively. Furthermore, SREBF2 and SREBF1 were highlighted in the day 14 causal reasoning network (Fig. 5C) and are required for cholesterol biosynthesis and uptake. Additionally, the SREBPs transcriptionally regulate genes ACAT2, HMGCR and HMGCS1 [62] which are differentially regulated (*p* ≤ 0.05 and |Log2FC |≥ 1.5) upon Anle138b treatment in both hAD tau seeded and unseeded RCNs at both day 7 and 14 (Table S1). Finally, pathways relating to cholesterol biosynthesis and metabolism were significantly enriched, particularly at day 14 (Table 2): For example, the “Superpathway of Cholesterol Biosynthesis” pathway had an adjusted *p*-value of 5.01E-16 based on DEGs obtained at day 14 (Supplementary File 3, Figure S3). Cholesterol homeostasis has been implicated in tau aggregation inhibition via pTau proteasomal degradation [63] and through perturbations in calcium signalling, which would modulate the activity of tau kinases as well as rescuing synaptic plasticity [64]. Moreover, cholesterol metabolism was found to modulate amyloid pathology, also observed with Anle138b treatment. Overall, this provides a plausible and consistent argument for the modulation of cholesterol homeostasis by Anle138b, in particular via the NPC1 target.

With respect to *inflammation*, Martinez Hernandez et al. observed an amelioration of inflammatory gene expression signatures following Anle138b treatment of a tau mouse model [8], which is consistent with the mediators of inflammation identified in the current study (NF-kB subunits NFKB1, NFKB2, RELA from target prediction; see Table 3, e.g. NFKB2 predicted probability of 0.62). Notably and in agreement with this, the structurally similar compound CHEMBL1567097 (see Fig. 6) was found to increase neuronal expression of NF-kB genes (at 0.64 μM, Table 4), and we identified subunits NFKB1 and RELA in day 7 and 14 causal reasoning networks, as well as other mediators of neuroinflammation NFATC4 and HMGB1 at day 3 (Fig. 5). We also identified significantly enriched pathways relating to neuroinflammation across all time points and in Anle138b-treated hAD tau seeded and unseeded RCNs (Figures S3 and S4). In particular, the interleukin pathway “IL-22 signalling pathway” was enriched with an adjusted *p*-value of 0.0178 at day 3, “IL-1 signalling pathway” with adjusted *p*-value of 0.0209 at day 7 and the “Neuroinflammation signalling pathway” with an adjusted *p*-value of 0.0141 at day 14 (Supplementary File 3). Neuroinflammation has previously been implicated in a ‘vicious cycle’ of AD progression, where it both increases tau pathology and is increased by tau pathology [65]; thus, Anle138b-mediated regulation of neuroinflammation could plausibly reduce tau aggregation.

Another potential mechanism of action of Anle138b could be *tau degradation*, either via the ubiquitin-proteasomal system or by autophagy. Predicted targets SENP6 and SENP8 (Table 3, probabilities of 0.57 and 0.59 respectively) modulate ubiquitylation [66], whilst RAB9A (Table 3, probability of 0.58) is involved in autophagy. With regard to experimental evidence, structurally similar compounds CHEMBL1567097 and CHEMBL1370387 activate RAB9A (at 0.71 and 1.27 μM respectively, Table 4). Additionally, ubiquitin/proteasome markers UBB, PSMD10, USP (and others) were found in the day 7 causal reasoning network and autophagy modulator ATF4 at day 3 (Fig. 5). It should be noted, however, that no relevant pathways were significantly enriched and that markers of ubiquitination were unchanged in Anle138b treated neurons and those of autophagy in Anle138b-treated mouse model [6]. As described above, this ubiquitination marker observation could be due to lack of tau pathology in the neurons used by Wagner et al. Nevertheless, the previous lack of experimental evidence for Anle138b-mediated changes in ubiquitination and autophagy decreases the confidence in this hypothesis.

Finally, we hypothesise other potential mechanisms of Anle138b-induced changes to *tau kinase activity*, which would reduce pTau and thus NFT formation. Anle138b inhibition of ALOX15 (Table 3, probability = 0.60) would mediate CDK5 and SAPK/JNK phosphorylation of tau [49], thus explaining why previous Anle138b reduction in pTau was observed despite no significant change in activity of GSK3β [6]. Additionally, transcription factors NFATC4 and ELK1 are downstream of SAPK/JNK and were identified in causal reasoning analysis as a possible mode of action of Anle138 (Fig. 5), as well as the “CDK5 signalling” pathway which would also be affected by ALOX15 modulation (Figure S4, adjusted *p*-value of enrichment = 0.0015 at day 7 in hAD tau seeded RCNs). Other hypotheses are inhibition of tau phosphorylation via the PI3K/AKT/GSK3b cascade—“PI3K/AKT” pathway was significantly enriched at day 3 in unseeded RCNs (FDR-adjusted *p*-value = 0.004, Figure S3). One entry point for this signalling pathway could be GPCRs such as δ-opioid and dopamine D_2_ receptors [67]—GPCR signalling pathways were significantly enriched at day 14 in both hAD tau seeded and unseeded RCNs (Table 2)—modulating calcium signalling [68] and the activities of calcium messenger CALR and other downstream proteins (e.g. MAPKs) in the causal reasoning-derived networks of Anle138b perturbation (Fig. 5). Alternatively, the PI3K/AKT/GSK3B pathway could be modulated by IGF1/IRS1 [41], where the insulin receptor IRS1 and other mediators of glucose metabolism such as HIF1A were identified via causal reasoning (Fig. 5). However, no predicted direct targets were found that were relevant for these two mechanisms, and Anle138b was found previously to not affect GSK3β phosphorylation [6]. Therefore, the ALOX15 mechanism is in our opinion the most compelling hypothesis of Anle138b-mediated tau kinase modulation.

In terms of mechanistic hypotheses generated from only one piece of analysis, we found CLK1 as a predicted target (probability = 0.52, 10 μM), which regulates the alternative splicing of tau protein [55] and significantly enriched pathways related to extracellular matrix degradation (e.g. “Degradation of the extracellular matrix”) were found in both hAD tau seeded and unseeded RCNs at day 14 (Table 2, Table S2). As mentioned previously, pharmacological reduction in hippocampal extracellular matrix has reversed early memory deficits associated with AD [28]. The lack of a consensus for these hypotheses over more than one analysis method do not necessarily mean that these findings are false; however, we have less confidence in them and would proceed with the consensus hypotheses first when conducting experimental validation.

## Limitations and considerations

It is important to note that the processes inferred to be modulated by Anle138b through this analysis may represent off-target or side effects (or not result in any meaningful phenotypic change) and not be mechanistically responsible for the observed reduction in tau aggregation. However, the current work provides testable hypotheses, based on experimental data, and are biologically plausible. In particular, the experiments that generated the transcriptomics data in this study were carried out using neuronal cells in vitro, and thus, the inferred changes in, e.g. cholesterol homeostasis and ALOX15 modulation would also need to be visible in vivo to represent a meaningful mode of action of Anle138b. In vitro gene expression measurements are not always concordant to in vivo measurements due to the pharmacokinetic (PK) properties of compounds which govern their absorption, distribution, metabolism and excretion (ADME) in a living organism, though cellular models are valuable to generate hypotheses to reduce, refine and replace animal studies [69].

PK properties also govern the concentration of the compound at the site of action and can be used to translate in vitro target prediction to in vivo target engagement through the knowledge of compound *C*_max_ (maximum concentration in blood or brain) and plasma protein binding (PPB). *C*_max_ and PPB can be used to compute the *C*_max_ (unbound) which is the maximum unbound concentration [70] of a compound available to bind to potential targets. Pharmacokinetic experiments carried out during Anle138b administration of 5 mg in mice found a *C*_max_ (maximum concentration) in the brain of 125 μM [71], but the compound’s PPB fraction is not in the public domain. Predictions from pkCSM [72] suggest a fraction unbound of 0.08, which would make the *C*_max_ (unbound) of Anle138b around 10 μM. This is within 1 log unit of the predicted activities obtained in this study (Table 3); thus, in vivo target engagement is plausible, assuming a dose of 5 mg in mice.

## Conclusions

In this work, we aimed to understand in more detail the molecular mechanisms of Anle138b, a tau aggregation inhibitor which exhibits efficacy in in vivo mouse models of AD. To this end, we utilised rat cortical neurons (RCN) seeded with hAD tau as a cellular model of tau aggregation in Alzheimer’s disease [15]. Cells were treated with Anle138b and gene expression changes were measured by RNA-Seq for three different treatment durations (of 3, 7 and 14 days) and compared to control. We used this data to perform pathway enrichment and causal reasoning and performed target prediction using the chemical structure of the compound. Through this investigation, we generated biologically plausible and testable molecular hypotheses for the putative mode of action of Anle138b, such as changes in cholesterol homeostasis mediated via NPC1 and SREBPs, mediation of neuroinflammation via interleukins and NF-kB and modulation of tau kinase activity via ALOX15. Experimental validation of these findings (which was outside the scope of the current study), including in vivo studies, would hence allow us to identify molecular mechanisms which lead to the modulation of Alzheimer’s disease in vivo and hence also raise the possibility to identify new chemical matter with the required bioactivity. On a methodological level, we demonstrated the utility of an integrated approach to understanding the mode of action of an experimental drug in an area of high medical need, with the approach being agnostic to particular chemical or therapeutic areas and hence generally applicable.

## Methods

### Preparation of hAD seeds

Post-mortem human brain tissues from AD patients were obtained from Manchester Brain Bank and King’s College London Neurodegenerative Diseases Brain Bank. Consent was obtained from legal guardian(s) for the involvement of Alzheimer’s disease patients (vulnerable population). The brain banks were responsible for obtaining informed consent from legal guardian(s). The Manchester Brain Bank Management Committee (University of Manchester) and the MRC London Neurodegenerative Disease Brain Bank (King’s College London) assessed the applications on their scientific merit and ethical use of tissues and granted approval for the specified use. All experiments were performed according to HTA (Human Tissue Authority) guidelines. Both Brain Banks have generic ethics committee approval to function as research tissue banks, which means that they can provide stem cells and tissue samples to UK-based researchers for a broad range of studies without the need for the researchers to obtain their own ethics approval. Post-mortem human brain tissue was obtained as described above after confirmation of consent and histological confirmation of tau pathology. All human tissue was handled in compliance with the regulations set by the HTA Human Tissue Act 2004.

The pool of cortical tissues used were obtained from ∼20 patients with Alzheimer’s disease, modified Braak (Brain Net Europe) stage 6 with moderate amyloid angiography. The average age was 71 years, and the gender was mixed male and female. The samples chosen for the preparation were those with the highest levels of AT8-positive Tau (0.5 μg/ml) as determined by AlphaScreen, described previously in [73]. Sample dilutions were incubated overnight at 4 °C with antibody-bound AlphaScreen acceptor beads (PerkinElmer) and antibody pair in 0.1% Casein in PBS. Following binding of the sample to the antibody pair (biotinylated antibody and acceptor-bead bound antibody) overnight, AlphaScreen donor beads (PerkinElmer) were added to the appropriate concentration and incubated at room temperature for 4 h before reading the plate on an Envision plate reader (PerkinElmer). The purification of tau from hAD brains followed a protocol previously described in [15] (adapted from [74]). For each purification, the tissue was thawed, the white matter was dissected out, and 100 g of cortical grey matter was homogenized using an Ultra Thurrax (IKA T25, 25,000 rpm, 10 min) in 400 ml of Dulbecco’s phosphate-buffered saline (DPBS) supplemented with complete protease inhibitor tablet (Roche) and centrifuged at 10,000 g for 10 min at 4 °C. The pellets were re-extracted twice using the same buffer conditions as the starting materials, and the supernatants from the three extractions were filtered through a Kim wipe and pooled. Thirty percent of sarkosyl was added to the pooled supernatant for a final 1% concentration. The sample was incubated in a glass bottle, shaking at room temperature for 1 h on a flat rotating shaker at a medium speed. After sarkosyl extraction, the sample was centrifuged at maximum speed (45,000 rpm, 158,000 g) for 60 min at 4 °C in an Optima XPN-80 ultracentrifuge. The resulting 1% sarkosyl-insoluble pellet was washed once in PBS/complete and then resuspended in Tris 50 mm, pH 7.4, containing complete (50 μl Tris/g grey matter) and sonicated with ∼20 1-s pulses (40% amplitude, Soniqa Q125 sonication probe) and named “hAD seed.” Subsequently, Tau concentration of the hAD seed was determined using AlphaScreen as described previously. When used to treat neurons, the hAD seed stock was diluted in media to the appropriate concentration.

### Rat cortical neuron treatment with hAD tau seed and RNA extraction

Rat cortical neurons (RCNs) were plated in 2 mL of media at 1.7 × 10^6^ cells/well in six 6-well plates (#354,413, Corning); four plates per time point were harvested at days in vitro (DIV) 3, 7 and 14; at DIV 3, cells were treated with 18 nM of hAD seed that was first sonicated for 1 min at 20% amplitude, with 1-s pulses (QSonicaProbe), and then filter sterilised with a 0.22-μM filter and syringe (PN4602, PalAcrodisc). At the same time, Anle138b at 8 µm or DMSO were added to the appropriate wells. After 5 h, the 6-well DIV3 plates were washed twice with DPBS and RNA extracted using Ambion RNAqueoskit (ThermoFisher, #AM1914). At DIV 7 and 14, the appropriate plates were extracted as described above. At DIV 14, a half media change was performed, and Anle138b or DMSO were replenished (8 μM final concentration); these plates were successively extracted after a week. The extracted RNA concentration was then determined with a NanoDrop spectrophotometer (Thermo Fisher), and its quality was assessed with Agilent (Agilent, RNA 600nano, 5067–511). All animal procedures were performed in accordance with the Animals (Scientific Procedures) Act 1986 and were reviewed by the internal Animal Welfare and Ethical Review Body (the Eli Lilly Animal Welfare Board) to ensure they comply with ethical and welfare standards. Procedures were in compliance with the ARRIVE guidelines.

### RNA-seq analysis

Raw RNA-seq reads were processed to obtain gene-level RNA-abundance values using a pipeline developed by Eli Lilly and Company. FASTQ files were checked for integrity and quality (base quality, base composition, heterologous organisms, rRNA/mitochondrial/viral and adaptor content) before aligning to the Rattus norvegicus reference genome (assembly Rnor_6.0) using GSNAP [75]. All samples passed additional, post-alignment quality control (3′ bias, flow cell bias, template length, sample relatedness, species). Quantification was performed at the exon level (using NCBI-based gene models) and Log2 median-exon counts used to denote gene-level RNA-abundance for 18,049 genes. Quantile normalisation across all gene-level summarised samples was performed. Unsupervised clustering approaches (PCA, hierarchical clustering based on Euclidean distance and Pearson correlation) did not identify any groupings associated with technical factors. A treatment by days in vitro (DIV) factorial model was used for differential expression analysis. There were three independent samples per treatment*DIV combination. Plate effects were handled by a mixed model when detected (Rlmer, R version 3.52 https://www.r-project.org/nosvn/pandoc/lme4.html); otherwise, a linear model was used (Rlm). Within gene FWER for pairwise treatment contrasts at each DIV level (i.e. Seed-Control@3, 7, or 14 DIV) were controlled by a Tukey procedure (Remmeans). The minimum Tukey corrected *p*-value within a gene was then used to control the false discovery rate to 5% across genes (Rp.adjust(,method = ”fdr”)). This produced a set of FDR adjusted *p*-values, one per gene. To resolve which of multiple contrasts within a gene were significant, both in terms of FWER and FDR, the maximum of the minimum FWER *p*-values was found for FDR adjusted *p*-values < 0.05 and called p*. FWER *p*-values less than p* identified significant contrasts overall. Fold change effect sizes from model predicted means were computed to aid summarisation of results.

### Enrichment analysis

Gene expression data as rat Entrez IDs were submitted for enrichment analysis using Ingenuity Pathway Analysis (IPA, QIAGEN Inc., http://digitalinsights.qiagen.com/Ingenuity/pathwayanalysis) ‘Core Analysis’ tool for each DIV [3, 7, 14] separately. Mappings to human were carried out automatically within the IPA pipeline. Significant genes were selected for downstream analysis if they satisfied the criteria |logFC|≥ 1.5 and adjusted *p* value ≤ 0.05. Additionally, enrichment analysis was carried out programmatically for GO [76] Biological Processes, MetaCore (Clarivate Analytics) pathway maps and networks using the CBDD R package (v. 9.1) [77] enrichment() function with ontology = pathway_onto, network_onto or go_onto, and Reactome [78] using ReactomePA (v 1.3) [79] enrichPathway(). Significant genes were selected according to the above thresholds. For the purpose of the programmatic enrichment analysis, the gene IDs were first converted from rat Entrez ID to human Entrez ID using the metabaser (Clarivate Analytics, v. 4.2.3) function get.gene.orthologs(to = ”human”,from = ”rat”). If one rat gene mapped to multiple human genes, then the measurement row was expanded to correspond to all human genes. If one human gene mapped to multiple rat genes, then the mean log2FC and *p*-values were taken as the human gene’s measurement. The human Entrez IDs were then converted to their corresponding HGNC gene symbols for enrichment analyses using the org.Hs.eg.db (v. 3.8.2) [80]annotation library with the AnnotationDBI (v. 1.48) [81] select(column = ”ENTREZID”, keytype = ”SYMBOL”) function. In all cases, enrichment background was set to every gene measured in the RNA-Seq experiments, representing the cell-specific transcriptome of the rat cortical neuron. Pathways were considered significantly enriched if the BH-adjusted *p*-values were ≤ 0.05. GO terms were further filtered for redundancy using REViGO [82] online server, submitted with their adjusted *p*-values with ‘Allowed similarity’ set to medium (0.7).

### AD associations

Open Targets 20.06 (last updated June 2020) [24] Tauopathies and Alzheimer Disease gene lists (HGNC symbols) were accessed and downloaded as a.csv file on 8 July 2020, to compare with the Anle138b-treated DEGs (after converting to HGNC symbols).

### Network data preparation

The OmniPath [83] PPI network was extracted from the CARNIVAL supplementary data with nodes as UniProt IDs. The IDs were converted to HGNC symbols using the org.Hs.eg.db annotation library with the AnnotationDBI select(column = ”UNIPROT”, keytype = ”SYMBOL”) function. MetaBase networks were extracted at three different confidence levels. The entire network was extracted using the get.globalnetwork (species = ”human”) function of metabaser v.4.2.3 (November 2019) and converted to a data frame. Edges that were not direct molecular interactions were removed. Network object IDs were then converted to corresponding Entrez IDs using the CBDD v. 9.1 function convertNetworkObjects2Entrez (networkobject, species = ”human”). Nodes which did not convert to Entrez ID were removed. If one network object corresponded to multiple Entrez IDs—i.e. a protein complex or family—it was assumed that all members of the protein complex take part in the interaction. Only signed edges were kept. For the PPI network, interactions with mechanism “co-regulation of transcription”,” Transcription regulation”, “Influence on expression”, “miRNA binding”, “Unspecified”, or “Pharmacological effect” were removed.

### Data preparation for causal reasoning

Data were prepared in different ways depending on the algorithm being used. The following were undertaken for data at days 3, 7 and 14. For CARNIVAL and CausalR, the RNA-seq data (as human HGNC symbols converted from rat Entrez IDs as described in the “Enrichment analysis” section) was transformed into transcription factor (TF) activities. Briefly, the most significant (*p* ≤ 0.05) genes were run with their log2FC values into DoRothEA [84], which uses a consensus TF-gene regulon to compute enrichment scores for transcription factors. For CARNIVAL, the same process was repeated with the PROGENy [85] pipeline to obtain pathway scores. The TF activities were used with CARNIVAL as-is, but for use in CausalR, were discretised where − 1 indicates inactivation, + 1 activation and 0 no change. For IPA causal reasoning analysis, the genes were subject to thresholding at the start of the pipeline os abs(logFC) ≥ 1.5 and *p* ≤ 0.05, the cut-off was carried out automatically as part of the pipeline after thresholds were defined.

### Causal reasoning

Causal reasoning analysis was run programmatically with two different algorithms (CARNIVAL [86] and CausalR [87]), two network sources (OmniPath [83] and MetaBase at different confidence thresholds) as well as the IPA GUI pipeline. CausalR RankTheHypotheses() function was run up to a path length of 5; the significant (*p* <  = 0.05) scored nodes were considered as hypotheses as output from CausalR. In addition, a subnetwork output was produced by finding “consensus nodes” (modified ScanR() function from [88]) which were proteins significantly scored across the greatest number of path lengths (1 to 5), then reconstructing their subnetworks from the nodes to the experimental input, via concordant interactions (WriteExplainedNodesToSifFile()). If multiple consensus nodes were found, then their subnetworks were combined into a “consensus subnetwork”, aiming to capture the cellular signalling pathways modified by Anle138b. Such subnetworks were also taken as output. CARNIVAL InvCarnival() function was run (no user-provided target nodes) with the TF enrichment scores and PROGENy pathway weights to optimise and reconstruct subnetworks. Finally, IPA causal reasoning analysis and upstream regulator analysis were run with default settings. The significant (*p* <  = 0.05) nodes from each of the URA (upstream regulator analysis) and CNA (causal network analysis) tables were taken as output from IPA analysis and were filtered further to only include nodes representing proteins (excluding compounds and other molecular entities from the analysis). All hypotheses across the different algorithms and networks were agglomerated into a table detailing the most commonly highlighted proteins overall and across different experiments and analysed further.

### Cheminformatics analysis

Target prediction [89, 90] was carried out for Anle138b using Python tool PIDGINv4 [91, 92] (available at http://github.com/BenderGroup/PIDGINv4). PIDGINv4 predict.py was run for the Anle138b SMILES with command-line options of –organism = “Homo sapiens, -b (bioactivity) = 0.1, 1, 10, 100 um, and applicability domain filter turned off (–ad 0). The matrix of confidence percentiles for each prediction was computed by re-running the command with the –percentile flag to infer the applicability domain of Anle138b with each model. Additionally, we ran the sim_to_train.py script to identify the most structurally similar compounds to Anle138b in the training set for each model.

## Supplementary Information

**Additional file 1.**  RNA-Seq results from Anle138b perturbation experiments on rat cortical neuron (RCN) cells, with their Log2FC and adjusted p-values.

**Additional file 2.** Full details of OpenTargets Tauopathies and Alzheimer's Disease association scores of each DEG in each experiment.

**Additional file 3.** All enriched pathways and their associated adjusted *p*-values.

**Additional file 4.** Upstream regulators inferred from causal reasoning across different algorithms/networks and the frequency of their occurrence.

**Additional file 5.** Full matrix of target predictions.

**Additional file 6.** **Figure S1.** Heatmap showing the differential expression (as log2-fold change) of significantly differentially expressed genes when comparing hAD tau seeded RCNs to unseeded RCNs (Seed - Control) and Anle138-treated hAD tau seeded RCNs to vehicle control hAD tau seeded RCNs (Anle + Seed - Seed) at DIV (days in vitro) 3. **Figure S2.** Heatmap showing the differential expression (as log2-fold change) of significantly differentially expressed genes when comparing hAD tau seeded RCNs to unseeded RCNs (Seed - Control) and Anle138-treated hAD tau seeded RCNs to vehicle control hAD tau seeded RCNs (Anle + Seed - Seed) at DIV (days in vitro) 7. **Figure S3.** Heatmap showing the differential expression (as log2-fold change) of significantly differentially expressed genes when comparing hAD tau seeded RCNs to unseeded RCNs (Seed - Control) and Anle138-treated hAD tau seeded RCNs to vehicle control hAD tau seeded RCNs (Anle + Seed - Seed) at DIV (days in vitro) 14. **Figure S4.** Most significantly enriched pathways at each time point in Anle138b-treated unseeded RCNs. Significance level of *p* = 0.05 is indicated as a red dashed line on the x-axis. **Figure S5.** Most enriched pathways at each time point in Anle138b-treated hAD Tau seeded RCNs. Significance level of *p* = 0.05 is indicated as a red dashed line on the x-axis. **Table S1.** DEGs overlapping with Open Targets (July 2020) Alzheimer’s Disease association list (**bold**) or Tauopathies association list (underlined) in each Anle138b perturbation experiment, as well as the overall overlap with AD + Tauopathies, and the corresponding Odd’s Ratio and *p*-value calculated with Fisher’s Exact Test. **Table S2.** Full list of overlapping pathways (FDR-adjusted *p*-value <= 0.05) between different Anle138b perturbation (hAD seeded or unseeded) RCN experiment at each time point. **Table S3.** Node list for each signalling subnetwork reconstructed from causal reasoning analysis of Anle138b transcriptomic data on unseeded and hAD tau seeded RCNs, bolded has prior disease association (from Open Targets). **Table S4.** Target prediction results from PIDGINv4 using the Anle138b chemical structure, predictions with AD (applicability domain) cutoff of 50 and probability cutoff of 0.3. Column definitions: Activity = bioactivity in uM, Probability = random forest probability of activity at stated threshold, ad = applicability domain percentile of the model, Nearest Neighbour ChEMBL ID = closest compound in the model training set, Similarity = Tanimoto similarity of the nearest neighbour, Alz Gene/Tau Gene = OpenTargets gene associations.

## Data Availability

The dataset(s) supporting the conclusions of this article are included within the article and its additional files. The MetaBase™ networks used in this study are available under a license from Clarivate™. You may not copy or re-distribute this material in whole or in part without the written consent of Clarivate™.

## Abbreviations

AD: Alzheimer’s disease
NFT: Neurofibrillary tangles
Tau: Microtubule-associated protein tau
hTau: Human tau
RCN: Rat cortical neuron
DMSO: Dimethyl sulfoxide
RNA-Seq: Ribonucleic acid sequencing
DEG: Differentially expressed gene
GPCR: G-coupled protein receptor
FDA: Food and Drug Administration
ECM: Extracellular matrix
PPI: Protein–protein interaction
pTau: Phosphorylated tau
