# Supplementary material for: Mechanism of action deconvolution of the small-molecule pathological tau aggregation inhibitor Anle138b
Source: Alzheimers Res Ther. 2023 Mar 14;15:52. doi: 10.1186/s13195-023-01182-0 (PMC10012450; doi:10.1186/s13195-023-01182-0)
Supplement: Supplementary file 6 — Additional file 6. Figure S1. Heatmap showing the differential expression (as log2-fold change) of significantly differentially expressed genes when comparing hAD tau seeded RCNs to unseeded RCNs (Seed - Control) and Anle138-treated hAD tau seeded RCNs to vehicle control hAD tau seeded RCNs (Anle + Seed - Seed) at DIV (days in vitro) 3. Figure S2. Heatmap showing the differential expression (as log2-fold change) of significantly differentially expressed genes when comparing hAD tau seeded RCNs to unseeded RCNs (Seed - Control) and Anle138-treated hAD tau seeded RCNs to vehicle control hAD tau seeded RCNs (Anle + Seed - Seed) at DIV (days in vitro) 7. Figure S3. Heatmap showing the differential expression (as log2-fold change) of significantly differentially expressed genes when comparing hAD tau seeded RCNs to unseeded RCNs (Seed - Control) and Anle138-treated hAD tau seeded RCNs to vehicle control hAD tau seeded RCNs (Anle + Seed - Seed) at DIV (days in vitro) 14. Figure S4. Most significantly enriched pathways at each time point in Anle138b-treated unseeded RCNs. Significance level of p = 0.05 is indicated as a red dashed line on the x-axis. Figure S5. Most enriched pathways at each time point in Anle138b-treated hAD Tau seeded RCNs. Significance level of p = 0.05 is indicated as a red dashed line on the x-axis. Table S1. DEGs overlapping with Open Targets (July 2020) Alzheimer’s Disease association list (bold) or Tauopathies association list (underlined) in each Anle138b perturbation experiment, as well as the overall overlap with AD + Tauopathies, and the corresponding Odd’s Ratio and p-value calculated with Fisher’s Exact Test. Table S2. Full list of overlapping pathways (FDR-adjusted p-value <= 0.05) between different Anle138b perturbation (hAD seeded or unseeded) RCN experiment at each time point. Table S3. Node list for each signalling subnetwork reconstructed from causal reasoning analysis of Anle138b transcriptomic data on unseeded and hAD tau seeded RCNs, [file 13195_2023_1182_MOESM6_ESM.docx]

**Mechanism of action deconvolution of the small-molecule pathological Tau aggregation inhibitor Anle138b**

# **Supplementary Figures**

#
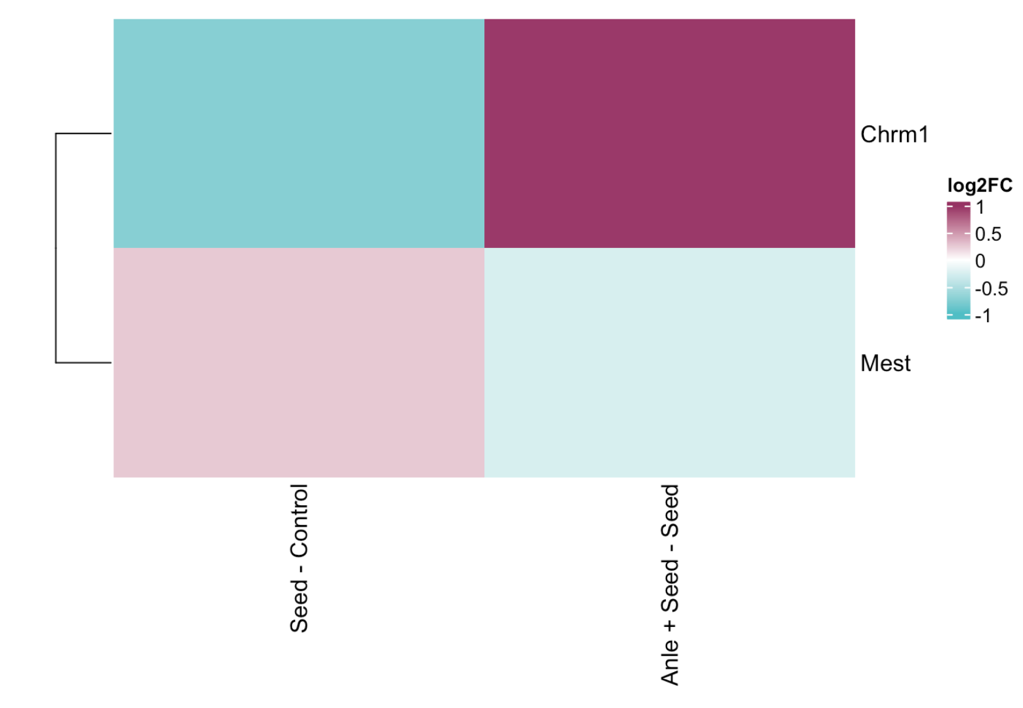


Figure S1: Heatmap showing the differential expression (as log2-fold change) of significantly differentially expressed genes when comparing hAD tau seeded RCNs to unseeded RCNs (Seed - Control) and Anle138-treated hAD tau seeded RCNs to vehicle control hAD tau seeded RCNs (Anle + Seed - Seed) at DIV (days in vitro) 3


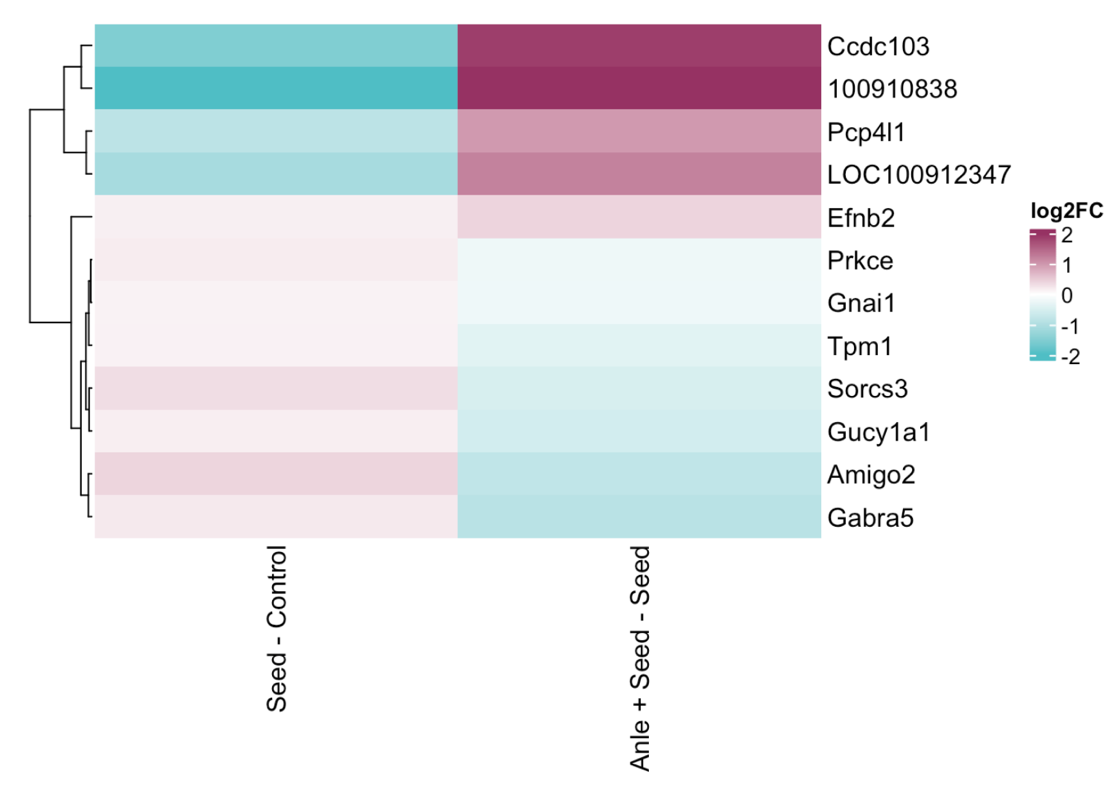


Figure S2: Heatmap showing the differential expression (as log2-fold change) of significantly differentially expressed genes when comparing hAD tau seeded RCNs to unseeded RCNs (Seed - Control) and Anle138-treated hAD tau seeded RCNs to vehicle control hAD tau seeded RCNs (Anle + Seed - Seed) at DIV (days in vitro) 7


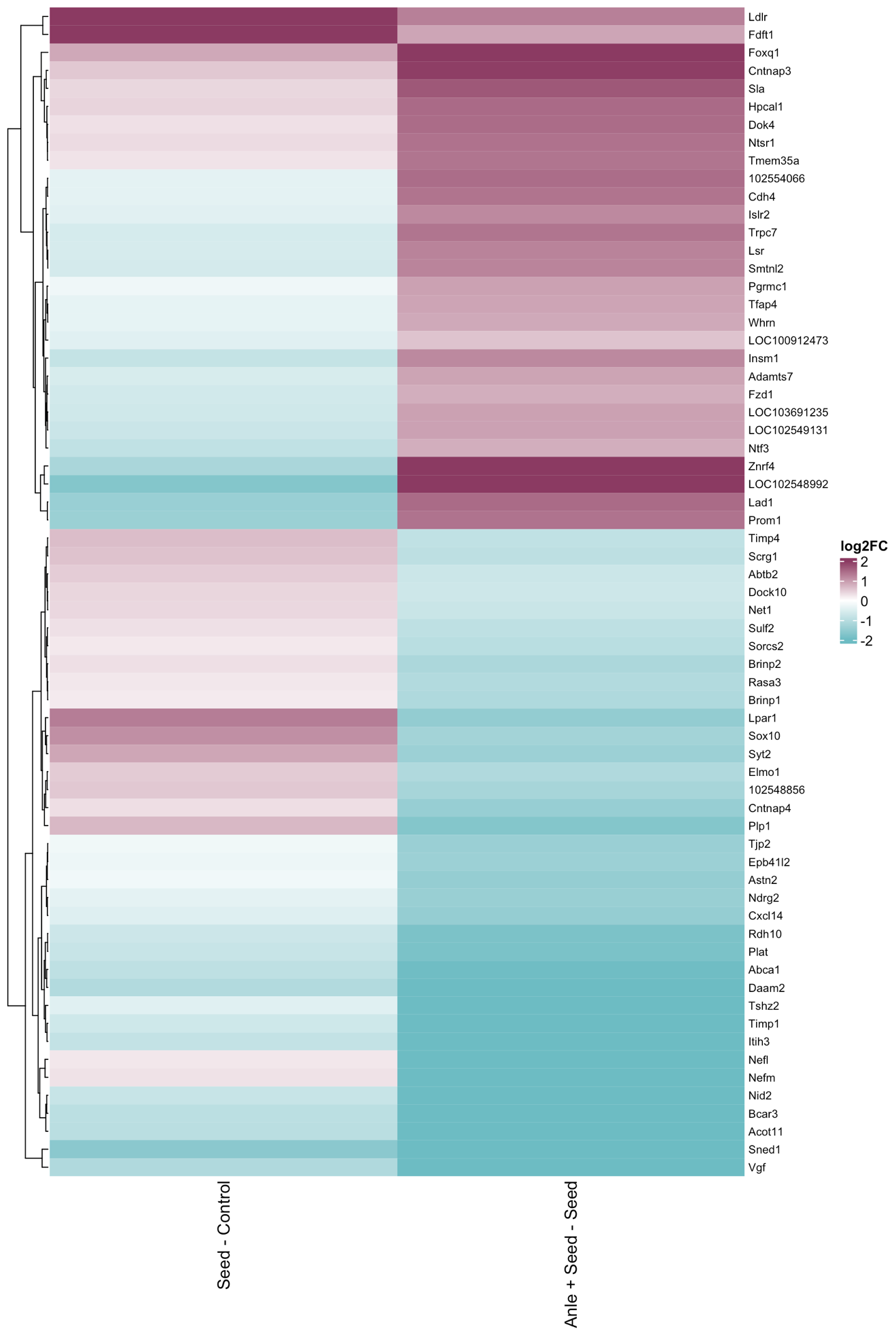


Figure S3: Heatmap showing the differential expression (as log2-fold change) of significantly differentially expressed genes when comparing hAD tau seeded RCNs to unseeded RCNs (Seed - Control) and Anle138-treated hAD tau seeded RCNs to vehicle control hAD tau seeded RCNs (Anle + Seed - Seed) at DIV (days in vitro) 14


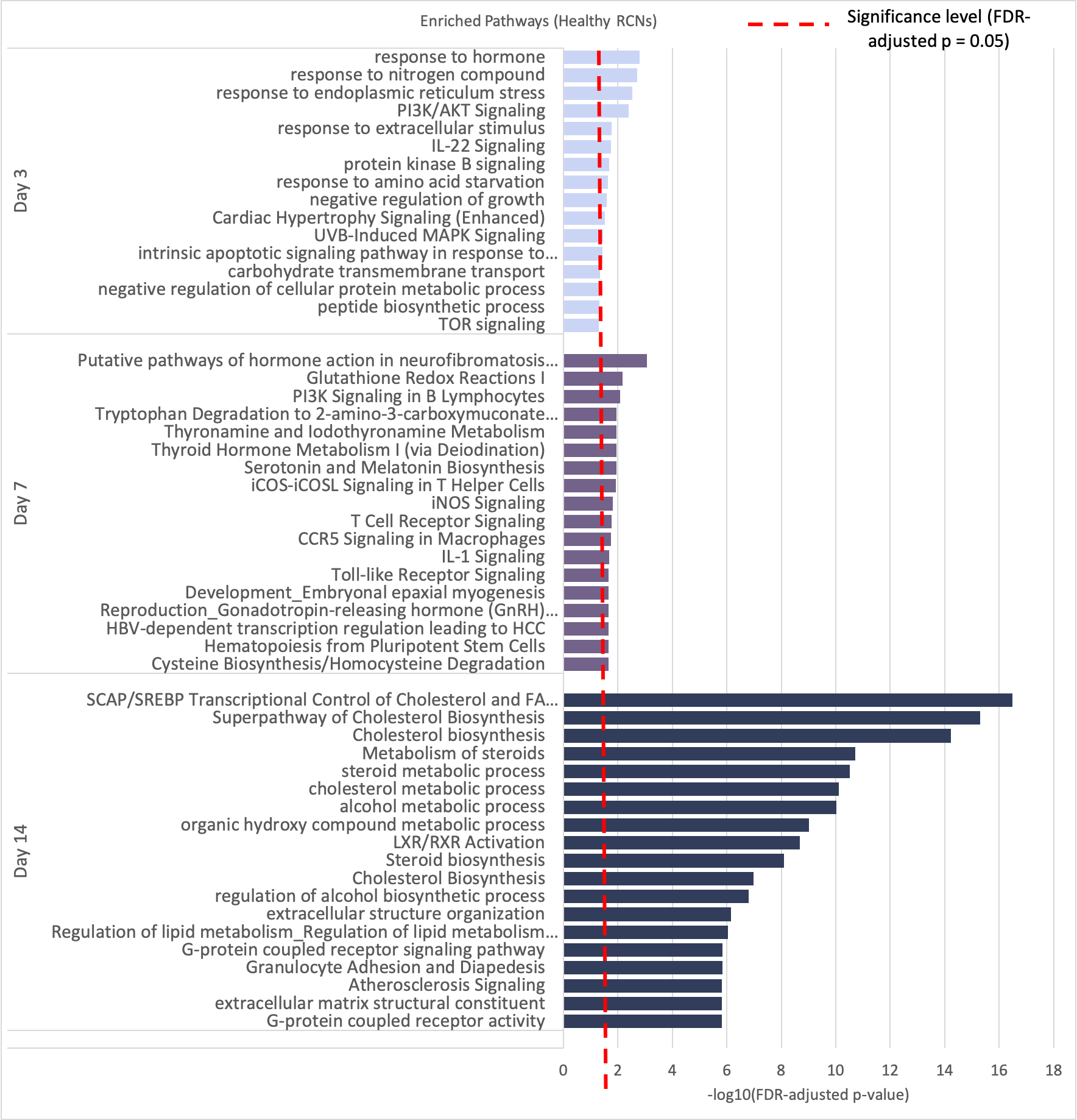


(Unseeded RCNs)

Figure S4: Most significantly enriched pathways at each time point in Anle138b-treated unseeded RCNs. Significance level of p = 0.05 is indicated as a red dashed line on the x-axis


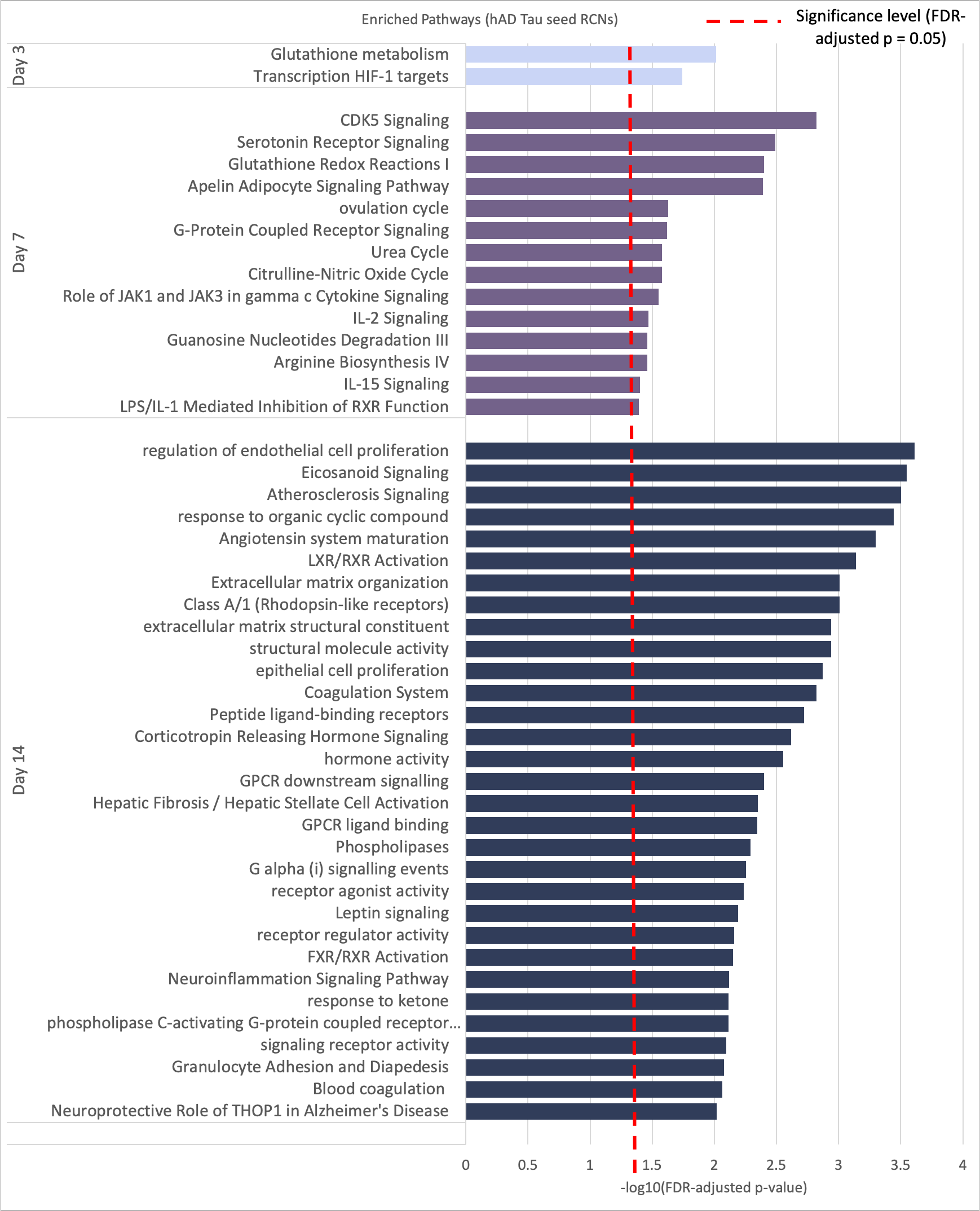


Figure S5: Most enriched pathways at each time point in Anle138b-treated hAD Tau seeded RCNs. Significance level of p = 0.05 is indicated as a red dashed line on the x-axis

# **Supplementary Tables**

Table S1: DEGs overlapping with OpenTargets (July 2020) Alzheimer’s Disease association list (**bold**) or Tauopathies association list (underlined) in each Anle138b perturbation experiment, as well as the overall overlap with AD + Tauopathies, and the corresponding Odd’s Ratio and p-value calculated with Fisher’s Exact Test

| **Time point (days)** | **Cell type** | **Overlap** | **Number of overlapping genes (AD + Tau)** | **Odd’s Ratio** | **Fisher’s Exact p-value** |
| --- | --- | --- | --- | --- | --- |
| 3 | Unseeded | **GDF15, TRIB3, EIF4EBP1, SESN2, HEYL** | 5 | 1.86 | 0.2706 |
|  | hAD tau Seeded |  | 0 | NA | NA |
| 7 | Unseeded | **ADRA1D, INPP5D, ESR1, CBS, NRGN, GPR6, GFAP, NGB, FOS, EGR1, SERPINF1, NAT2, MOBP, CD4, ARC, TPH2, GPX3, ASS1, HMGCS2, ME3, ANGPTL4, ATF3, CARTPT, PNLIP, GLI1, MCHR1, POSTN, EGR4, ADAMTS12,** FLNC | 30 | 1.14 | 0.3316 |
|  | hAD tau Seeded | **CAPN1, ADRA1D, ESR1, ADAMTS1, PLAC8L1, GFAP, HTR4, NGB, SYK, EGR1, SERPINF1, GRM2, GPX3, ASS1, DOCK5, NR4A1, EDNRA, CLDN11, HMGCS2, ANGPTL4, POSTN, GDA, EGR4, MFSD4A** | 24 | 1.14 | 0.3636 |
| 14 | Unseeded | **APOE, CLU, ACHE, HMGCR, GRIN2C, ADRA1D, SCN7A, INPP5D, APOC1, ABCA1, PDE7B, ESR1, PDGFRB, ADAMTS1, AQP4, RFX4, MT1M, MDGA1, CTSC, PLA2G7, NRGN, GPR6, RASGRF1, ADAMTS5, RSPO1, ZCWPW1, GFAP, HTR4, BDNF, SST, NGFR, TPSG1, DHCR24, CP, CALB2, ONECUT2, A2M, CYP2D6, S100B, TIMP1, ALB, VGF, VIM, NEFL, LDLR, CAV1, MT3, BACE2, TCN2, SELENOP, PLTP, LGALS3, ICAM1, FOS, TRPC6, GPR3, IGFBP7, CEBPD, NDRG2, GSTM3, CD34, HP, VIP, RGS4, CNTN2, SREBF2, SLC40A1, AEBP1, SYK, NEFM, GPNMB, PLAT, IL34, EZR, IL1RAP, GAD1, APLN, CADM1, SERPINF1, PHEX, BMP6, NT5C1A, GRPR, ALDH1A1, SLCO2A1, SPARC, PLCD1, TMEM97, NFATC2, COLEC12, ARC, GRM2, TPH2, HPCAL1, PON2, PPP1R1B, TNNI3, RXFP1, LOXL1, HDAC9, EDA, CDKN2B, FCGRT, SREBF1, PLD2, BGN, FOLH1, SLC29A1, SCG2, ECRG4, ASS1, PLA2G5, PROKR2, SLC5A7, RAPGEF3, EMP1, ZBTB20, NR4A1, SHMT1, HOPX, TES, SYNJ2, CTSZ, EDNRA, DGKB, FXYD1, LSS, LCAT, NEUROD1, AGT, CPM, NPR3, DYSF, HSPB6, SV2C, CCKBR, RAB27A, HMGCS1, OXTR, PRODH, EFEMP1, CTSH, HRH1, KCNH1, TCFL5, SORBS3, ITGB3, PNLIP, SPTA1, CYP51A1, UGT1A1, FOXO4, KCNQ3, C1S, CA7, GCK, MVD, GLI1, TUBB6, H2BC12, ADAMTS9, BHLHE22, ADAMTS14, TAGAP, ITGA1, ADGRD1, SLC39A12, C21orf91, KCNJ10, GRAMD2B, PBXIP1, KLF9, PCP4, HPCA, PPFIBP1, NKAIN4, DOCK5, LAMB2, GPR37L1, AHNAK, KLF15, ZFP36L2, ITGA7, ANXA11, ACSS1, PREX2, CD9, PAQR6, SLC25A18, DAAM2, SAMD4A, MLC1, LYNX1, KCNJ16, CARTPT, ERBB3, TRIL, FAM107A, RAB33A, NSDHL, SLC14A1, PTPRO, PLAAT3, S1PR3, ATP1A2, ID3, CBLN2, PAM, CSRP1, FAM126A, KCNA1, GAS1, MICALL2, SQLE, CXCL14, OLFM3, SFRP2, PCDH8, LGALS1, CHRDL1, EGR4, TTYH2, ACAT2, IDI1, DUSP5, C1orf115, TMEM35A, CCDC184, GPC3, ACVR1C, CLDN10, ZCCHC12, HSD17B11, CDH12, PDYN, FBXO32, RND3, PMP22, NRIP1, GPR85,** NHLH2, NTSR1, PPP1R3G, CYP4F2, ACOT11, CCDC80, STARD4, ACLY, NR4A3 | 248 | 1.44 | 0.0001714 |
|  | hAD tau Seeded | **C21orf91, KCNJ10, LPAR1, KLF9, PCP4, HPCA, DOCK5, GPR37L1, BCAS1, CA10, CLDN11, PREX2, CD9, PAQR6, DAAM2, LYNX1, KCNJ16, CARTPT, FAM107A, RAB33A, SLC14A1, PLAAT3, ID3, KCNA1, ST18, MICALL2, SLCO4A1, OLFM3, PCDH8, PPL, EGR4, MFSD4A, TRIM47, ACAT2, DUSP5, ANKRD34C, DUSP6, GPC3, ACVR1C, SOX8, ZCCHC12, PMP22, NRIP1,** NHLH2, ACOT11, NR4A3, **ACHE, GRIN2C, SCN5A, ADRA1D, SCN7A, INPP5D, ABCA1, ESR1, ADAMTS1, MT1M, GMNC, NRGN, GPR6, RASGRF1, ADAMTS5, RSPO1, GFAP, BDNF, SST, NGFR, CALB2, A2M, CYP2D6, TIMP1, VGF, THBD, NEFL, BACE2, APOD, ICAM1, FOS, LOX, IGFBP7, CEBPD, CD34, VIP, RGS4, PLP1, SLC40A1, AEBP1, NEFM, PLAT, GAD1, BMP4, SERPINF1, BMP6, ALDH1A1, THBS1, FASLG, NFATC2, COLEC12, ARC, GRM2, TPH2, MSTN, TNS3, TNNI3, RXFP1, HDAC9, KCNIP4, SREBF1, PLD2, SCG2, ECRG4, GNA14, PLA2G5, PROKR2, SLC5A7, EMP1, ZBTB20, GYPC, NR4A1, SHMT1, HOPX, SYNJ2, DGKB, LCAT, AGT, CPM, CRYAB, NPR3, SSTR3, DYSF, HSPB6, SV2C, UGT8, CCKBR, HMGCS1, EFEMP1, CTSH, KCNH1, TCFL5, ITGB3, PNLIP, KCNQ3, CA7, GCK, MVD, GLI1, TUBB6, BHLHE22, ITGA1, ADGRD1** | 149 | 1.28 | 0.02247 |

Table S2: Full list of overlapping pathways (FDR-adjusted p-value <= 0.05) between different Anle138b perturbation (hAD seeded or unseeded) RCN experiment at each time point

| **Experiment** | **Experiment** | **Overlapping Pathways** |
| --- | --- | --- |
| hAD tau seeded, Day 14 | Unseeded RCN, Day 14 | GPCR ligand binding, G alpha (i) signalling events, G alpha (q) signalling events, GPCR downstream signalling, Peptide ligand-binding receptors, Class A/1 (Rhodopsin-like receptors), Signaling by GPCR, Extracellular matrix organization, Degradation of the extracellular matrix, Neuroactive ligand-receptor interaction, LPS/IL-1 Mediated Inhibition of RXR Function, Zymosterol Biosynthesis, Superpathway of Geranylgeranyldiphosphate Biosynthesis I (via Mevalonate), Antioxidant Action of Vitamin C, Mevalonate Pathway I, GP6 Signaling Pathway, Superpathway of Cholesterol Biosynthesis, Neuroprotective Role of THOP1 in Alzheimer's Disease, Granulocyte Adhesion and Diapedesis, Neuroinflammation Signaling Pathway, FXR/RXR Activation, Phospholipases, Hepatic Fibrosis / Hepatic Stellate Cell Activation, LXR/RXR Activation, Atherosclerosis Signaling, Eicosanoid Signaling, regulation of endothelial cell proliferation, response to organic cyclic compound, extracellular structure organization, neurotransmitter biosynthetic process, positive regulation of lipase activity, isoprenoid metabolic process, phospholipase C-activating G-protein coupled receptor signaling pathway, extracellular matrix organization, G-protein coupled receptor signaling pathway, regulation of lipase activity, G-protein coupled receptor signaling pathway, coupled to cyclic nucleotide second messenger, neuropeptide signaling pathway, response to ketone, epithelial cell proliferation, lipid biosynthetic process, hormone activity, structural molecule activity, extracellular matrix structural constituent, receptor regulator activity, molecular transducer activity, receptor agonist activity |
| hAD tau seeded, Day 7 | Unseeded RCN, Day 7 | Glutathione Redox Reactions I, Citrulline-Nitric Oxide Cycle, Urea Cycle, Arginine Biosynthesis IV |
| hAD tau seeded, Day 3 | Unseeded RCN, Day 3 | - |

Table S3: Node list for each signalling subnetwork reconstructed from causal reasoning analysis of Anle138b transcriptomic data on unseeded and hAD tau seeded RCNs, bolded has prior disease association (from OpenTargets)

| **Table** | **Experiment** | **hAD Tau Seeded** | **Unseeded** | **Both** |
| --- | --- | --- | --- | --- |
| **A** | All | PRKAA2, **MAPK3**, **CREBBP**, **AKT1**, **ID2**, **HTT**, **NR4A1**, AXIN1, **SP1**, **AR**, **CALR**, **ATF3**, **SOX9**, **ATF4**, PAX3, SFTPA1, CRTC2, **IRS1**, HES1, MTA1, **MACF1**, TCF4, **PITX3**, **REST**, **RELB**, ZBED3, **ESRRA**, **SREBF2** | **SUMO1**, **RB1**, **GSK3B**, **ESR1**, MAPK11, **MAPK12**, **MAPK13**, **MAPK14**, **MAPK1**, CSNK2A1, **KAT2B**, **EP300**, NFKB1, RELA, **SOX2**, **TCL1A**, CDC27, **NR3C1**, **FOS**, NCOA3, FOXM1, **PARP1**, **FOXO1**, **HDAC1**, **AHR**, **E2F1**, **FOXK2**, KDM5B, **EGR1**, **NRF1** | **YAP1**, **MDM2**, **TP53**, **UBC**, **UBB**, **PPARGC1A**, SMAD3, **CTNNB1**, **CDK2**, **NR1H3**, **STAT1**, ELK1, **APEX1**, **CREB1**, **JUN**, **STAT3**, **SOX10**, **HIF1A**, SMARCA4 |
| **B** | Day 3 | **UBC**, **UBB**, **YBX1**, RNF121, TRIM23, **ATF4**, COMMD1, TRIM71, **BRD4**, **HIF1A**, RUNX3, KDM2B, PDLIM2, **RNF146**, **TRIB3**, DACH1, **TRIM36**, RNF216, MARCH4, **CDK9**, G2E3, FBXO42, USP6, CBLL2, **HECW1**, MARCH6, BFAR, **TMEM129**, RNF133, MSL2 | **TP53**, **RB1**, **ESR1**, RBFOX2, **HMGB1**, BRD9, CMA1, **CPEB4**, **NR3C1**, **FOS**, **F2**, PAX8, HMGA2, FOXM1, **SOX10**, **OLIG1**, **FOXO1**, **CDK6**, **SOX5**, SMARCA4, BICRA, NFATC4, **PSMD10**, FBXO21, DEPDC1, FCN2, **EID1**, ARID4A, **SP7**, **PBRM1**, ARMCX3, BLCAP, BICRAL, PPP1R26, MASP2, FGL2, **KLF1** | - |
| **C** | Day 7 | **MDM2**, **SUMO1**, **PARK7**, PRKD1, **CREBBP**, **PPARGC1A**, **ID2**, **BRCA1**, **NR4A1**, AXIN1, **SIRT6**, **CDK2**, **YY1**, **SP1**, ELK1, **STUB1**, **PSMA7**, BARD1, CDKN1B, **KCNIP3**, SFTPA1, CRTC2, **STAT3**, **IRS1**, KPNA4, HES1, MTA1, PIAS2, **FOXK2**, RBBP8, **OLIG2**, **IL10RA** | **YAP1**, **CASP7**, PRKACA, MAPK11, **MAPK13**, **MAPK14**, **KAT2B**, SMAD3, ARNT, IRF3, NFKB1, **CUL1**, UBA1, **WT1**, **NR1H3**, **PKM**, CDC27, **XIAP**, NCOA3, **ATF2**, **JMJD6**, FOXM1, **HIF1A**, KMT2A, CBFA2T3, **E2F1**, SOX4, **EGR1** | **APEX1**, **CREB1**, **JUN**, **XBP1** |
| **D** | Day 14 | **MDM2**, SIAH2, **MAPK3**, SMAD3, **ID3**, **ATM**, ELK1, **ATF3**, **DCLRE1C**, PAX3, **ATF2**, **COPS6**, **EWSR1**, **FOXO4**, SUB1, SNRPA, SOX4, **SREBF2** | **ESR1**, PRKAA1, MAPK11, **MAPK12**, **HSPA5**, CSNK2A1, PRKCI, CSNK2A2, GATA3, STK4, **SP1**, **PAK2**, RELA, **STAT1**, IGF2BP1, **TCL1A**, **S100A2**, MAP3K7, **CUEDC2**, **CSNK2B**, **MITF**, RASSF1, **JUN**, **SOX10**, **TRPV1**, **PARP1**, SMARCA4, **FOXK2**, **CLOCK**, MAF, **NRF1**, **MEF2A** | PRKAA2, **MAPK14**, AXIN1, **NR1H3**, **SREBF1**, **FOXO3**, **CREB1**, **REST**, **AHR**, **HIF1AN** |

Table S4: Target prediction results from PIDGINv4 using the Anle138b chemical structure, predictions with AD (applicability domain) cutoff of 50 and probability cutoff of 0.3. Column definitions: Activity = bioactivity in uM, Probability = random forest probability of activity at stated threshold, ad = applicability domain percentile of the model, Nearest Neighbour ChEMBL ID = closest compound in the model training set, Similarity = Tanimoto similarity of the nearest neighbour, Alz Gene/Tau Gene = OpenTargets gene associations

| **Symbol** | **Activity**  **uM** | **Probability** | **ad** | **Nearest Neighbour ChEMBL ID** | **Similarity** | **Alz Gene** | **Tau**  **Gene** |
| --- | --- | --- | --- | --- | --- | --- | --- |
| NFKB2 | 10  100  1 | 0.62  0.61  0.51 | 72  72  75 | CHEMBL1567097 | 0.50 | N | N |
| ALOX15 | 10  100  1 | 0.60  0.46  0.31 | 66  72  61 | CHEMBL1567097  CHEMBL239677 | 0.50  0.23 | Y | Y |
| SENP8 | 100  10 | 0.59  0.40 | 76  82 | CHEMBL1370387 | 0.45 | N | N |
| RELA | 1  100  10 | 0.58  0.52  0.42 | 71  69  69 | CHEMBL1567097 | 0.50 | N | N |
| RAB9A | 100 | 0.58 | 57 | CHEMBL1567097 | 0.50 | Y | N |
| NPC1 | 100 | 0.56 | 58 | CHEMBL1567097 | 0.50 | Y | Y |
| SENP6 | 100 | 0.56 | 79 | CHEMBL1370387 | 0.45 | N | N |
| NFKB1 | 1  10  100 | 0.52  0.52  0.52 | 73  68  68 | CHEMBL1567097 | 0.50 | N | N |
| ATAD5 | 100 | 0.52 | 99 | CHEMBL1567097 | 0.50 | N | N |
| CLK1 | 10  1  100  0.1 | 0.52  0.51  0.45  0.44 | 77  91  77  82 | CHEMBL2392365 | 0.41 | Y | Y |
| CFTR | 1 | 0.51 | 57 | CHEMBL4170137 | 0.27 | Y | Y |
| NCOA2 | 10 | 0.50 | 81 | CHEMBL1321356 | 0.34 | N | N |
| PRKD1 | 10  100 | 0.47  0.34 | 74  74 | CHEMBL1683314 | 0.36 | N | N |
| SENP7 | 100  10 | 0.46  0.39 | 77  76 | CHEMBL1370387 | 0.45 | N | N |
| TARDBP | 100 | 0.46 | 68 | CHEMBL1439442 | 0.43 | Y | Y |
| XDH | 1  10  100 | 0.45  0.35  0.31 | 70  72  66 | CHEMBL4095244 | 0.30 | N | N |
| NR2E3 | 100  10 | 0.45  0.36 | 81  87 | CHEMBL1358911 | 0.28 | N | N |
| CLK4 | 10  100 | 0.44  0.35 | 82  62 | CHEMBL2392365 | 0.41 | N | N |
| DYRK2 | 10  100  0.1  1 | 0.44  0.39  0.38  0.36 | 77  79  61  83 | CHEMBL3193172  CHEMBL1802358  CHEMBL3193172 | 0.27  0.26  0.27 | N | N |
| NCOA1 | 10 | 0.41 | 84 | CHEMBL1321356 | 0.34 | Y | Y |
| SMN1 | 100 | 0.41 | 65 | CHEMBL1567097 | 0.50 | Y | Y |
| SMN2 | 100 | 0.41 | 65 | CHEMBL1567097 | 0.50 | Y | Y |
| GFER | 100 | 0.40 | 67 | CHEMBL3192164 | 0.35 | Y | Y |
| ACVR1 | 10  100 | 0.38  0.37 | 84  85 | CHEMBL2004515 | 0.36 | N | N |
| GSK3A | 0.1  10  100  1 | 0.38  0.33  0.33  0.32 | 66  62  62  63 | CHEMBL2064531  CHEMBL2004515  CHEMBL2064531 | 0.33  0.36  0.33 | Y | Y |
| ALOX5 | 1 | 0.37 | 63 | CHEMBL303524 | 0.34 | Y | Y |
| GLP1R | 100 | 0.37 | 77 | CHEMBL1448722 | 0.47 | Y | Y |
| HTR5A | 1  100  10 | 0.36  0.31  0.32 | 64  60  65 | CHEMBL3770342  CHEMBL2429890 | 0.21  0.23 | N | N |
| CCR6 | 100 | 0.36 | 82 | CHEMBL1506728 | 0.32 | Y | Y |
| PPP1CA | 100 | 0.36 | 81 | CHEMBL1334407 | 0.23 | Y | N |
| CACNA1F | 10 | 0.35 | 95 | CHEMBL4074268 | 0.24 | N | N |
| PRNP | 10 | 0.35 | 78 | CHEMBL1380303 | 0.25 | Y | Y |
| HTR2B | 0.1 | 0.35 | 88 | CHEMBL3815066 | 0.31 | N | N |
| HIF1A | 1 | 0.34 | 99 | CHEMBL253429 | 0.31 | Y | Y |
| BRCA1 | 100 | 0.33 | 66 | CHEMBL1370897 | 0.37 | Y | Y |
| IDO1 | 10 | 0.33 | 65 | CHEMBL382312 | 0.38 | Y | Y |
| ALOX12 | 100 | 0.33 | 77 | CHEMBL1567097 | 0.50 | Y | Y |
| TGFBR1 | 10  100 | 0.33  0.30 | 56  57 | CHEMBL226838 | 0.35 | Y | N |
| CACNA1D | 10 | 0.33 | 95 | CHEMBL4074268 | 0.24 | Y | Y |
| ASIC3 | 100 | 0.32 | 94 | CHEMBL1770739 | 0.18 | N | N |
| DYRK1A | 100 | 0.32 | 62 | CHEMBL2392365 | 0.41 | Y | Y |
| CDK5 | 100 | 0.32 | 55 | CHEMBL2004515 | 0.36 | Y | Y |
| CCNA1 | 10  1 | 0.31  0.31 | 52  58 | CHEMBL204610  CHEMBL204087 | 0.30  0.29 | N | N |
| CDK5R1 | 100 | 0.31 | 54 | CHEMBL1802358 | 0.26 | Y | Y |
| GAPDH | 100 | 0.31 | 80 | CHEMBL69085 | 0.33 | Y | Y |
| MPI | 10 | 0.31 | 79 | CHEMBL1334768 | 0.36 | Y | Y |
| SGK1 | 10 | 0.30 | 68 | CHEMBL3356034 | 0.21 | Y | Y |
| GSK3B | 0.1 | 0.30 | 53 | CHEMBL2391121 | 0.31 | Y | Y |
| CLK2 | 10 | 0.30 | 77 | CHEMBL2392365 | 0.41 | Y | Y |
